# Identification of DNA methylation changes associated with disease progression in subchondral bone with site-matched cartilage in knee osteoarthritis

**DOI:** 10.1038/srep34460

**Published:** 2016-09-30

**Authors:** Yanfei Zhang, Naoshi Fukui, Mitsunori Yahata, Yozo Katsuragawa, Toshiyuki Tashiro, Shiro Ikegawa, Ming Ta Michael Lee

**Affiliations:** 1 Laboratory for International Alliance on Genomic Research, Center for Integrative Medical Sciences, RIKEN, Yokohama, Japan; 2 Genomic Medicine Institute, Geisinger Health System, Danville, PA, USA; 3 Clinical Research Center, National Hospital Organization Sagamihara Hospital, Kanagawa, Japan; 4 Department of Life Sciences, Graduate School of Arts and Sciences, the University of Tokyo, Tokyo, Japan; 5 Laboratory for Pharmacogenomics, Center for Integrative Medical Sciences, RIKEN, Yokohama, Japan; 6 Department of Orthopaedic Surgery, Center Hospital of the National Center for Global Health and Medicine, Tokyo, Japan; 7 Department of Orthopaedic Surgery, Tokyo Yamate Medical Center, Tokyo, Japan; 8 Laboratory for Bone and Joint Diseases, Center for Integrative Medical Sciences, RIKEN, Tokyo, Japan; 9 Institute of Biomedical Sciences, Academia Sinica, Taipei, Taiwan

## Abstract

Subchondral bone plays a key role in the development of osteoarthritis, however, epigenetics of subchondral bone has not been extensively studied. In this study, we examined the genome-wide DNA methylation profiles of subchondral bone from three regions on tibial plateau representing disease progression using HumanMethylation450 BeadChip to identify progression associated DNA methylation alterations. Significant differential methylated probes (DMPs) and differential methylated genes (DMGs) were identified in the intermediate and late stages and during the transition from intermediate to late stage of OA in the subchondral bone. Over half of the DMPs were hyper-methylated. Genes associated with OA and bone remodeling were identified. DMGs were enriched in morphogenesis and development of skeletal system, and HOX transcription factors. Comparison of DMGs identified in subchondral bone and site-matched cartilage indicated that DNA methylation changes occurred earlier in subchondral bone and identified different methylation patterns at the late stage of OA. However, shared DMPs, DMGs and common pathways that implicated the tissue reparation were also identified. Methylation is one key mechanism to regulate the crosstalk between cartilage and subchondral bone.

Osteoarthritis (OA) has generally been considered a degenerative disease that affects all of the joint structures, not only the cartilage, but also the subchondral bone, the synovium and joint capsular tissues. Although the degeneration of cartilage is the main feature of OA, increasing evidence has shown that the underlying subchondral bone plays a critical role in the initiation and progression of the disease^1^,^2^,^3^. During OA progression, the architecture and components of the subchondral bone are modified through the processes of remodeling by osteoblasts and osteoclasts to adapt to the altered mechanical loading^2^. DNA methylation is one common epigenetic mechanism for regulation of gene expression, chromosome stability, embryonic development, carcinogenesis and diseases. Previous studies using candidate gene strategy have identified that DNA methylation is one important mechanism in bone remodeling. Studies using primary osteoblasts, osteocytes and cell lines have confirmed that methylation in promoter regions or the vicinity of transcription start sites of *ALPL*, *SOST*, *RANKL* and *OPG*, which participate in OA, were associated with their low expression. Demethylation of these genes could increase their expression^4^,^5^,^6^, suggesting an important role of DNA methylation in osteogenesis.

Due to the difficulties associated with accessing the specific regions of subchondral bone specimens and subsequent isolation of adequate quantity and quality of DNA, most genome-wide DNA methylation studies were conducted on the cartilage. Hitherto, only two studies were conducted on bones, both of which were in the hip^7^,^8^. We previously reported a unique model system to obtain site-matched cartilage and subchondral bone specimens, which includes three regions to encompass the early, intermediate and late stage of OA^9^. Briefly, the outer region of the lateral tibial plateau (oLT) exhibited macroscopically normal cartilage; the inner region of lateral tibial plateau (iLT) showed intermediate erosion and the inner region of medial tibial plateau (iMT) had the most severe erosion of the cartilage (Supplementary Figure 1A). It has been demonstrated that this system could be used to study the progression associated gene expression and methylation^10^,^11^,^12^.

For this study, we aimed to evaluate genome-wide DNA methylation profiles in subchondral bone and identify the differential methylated genes (DMGs) and pathways associated with disease progression. Thus, we adopted the same model system and used the same array based methylation profiling in 12 knee joints with primary OA, of which DNA methylation profiles have been previously examined in the site-matched cartilage^12^. Furthermore, DMGs identified from the subchondral bone and cartilage were analyzed and compared to evaluate the common pathways and interactions during OA progression.

## Results

### Identification of differential methylated probes (DMPs) associated with OA progression in subchondral bone

Probe filtering and normalization were conducted together with the processing of cartilage methylation profile as the same batch of methylation chips were used^12^. PCA plot indicated no outliers, and the samples were specifically clustered by gender (supplementary Figure 2A). In the subsequent probe-filtering step, probes on chromosome X and Y were filtered out in order to eliminate the sex difference. A total of 472,958 probes (97.4%) were included in the analysis. Probes in *HOXA3*, *HOXA5* and *HOXA9* were selected for validation of methylation chip results using bisulfite Sanger sequencing as these probes covered high, intermediate and low methylation levels. Strong correlation was found between the two methods (r^2^ = 0.8188, p < 2.2*10^−16^. Individual p values are 0.00734, 5.1*10^−6^ and 0.00102 for *HOXA3*, *HOXA5* and *HOXA9*, respectively. Supplementary Figure 2B), suggesting the high credibility of the methylation chip data.

**Figure 1:**
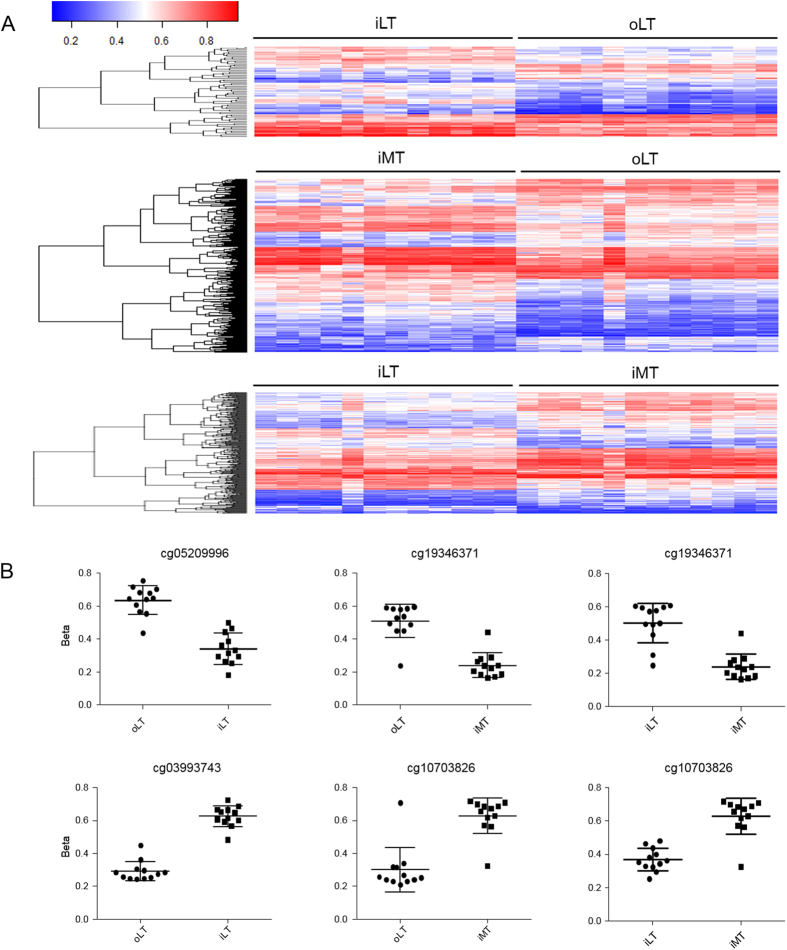
Genome-wide DNA methylation profiling in subchondral bone of knee OA. (**A**) Heat map of identified significant differentially methylated probes (DMPs) in iLT/oLT and iMT/oLT comparison groups. The methylation scale was shown on the top (0 = no methylation, 1 = 100% methylation). Horizontal axis: samples from the oLT, iLT and iMT region. Vertical axis: the dendrogram showing the clusters of DMPs. (**B**) β value plot of selected top DMPs in the iLT/oLT, iMT/oLT and iMT/iLT groups. Cg05209996 did not map to a gene. Cg03993743, cg19346371 and cg10703828 mapped *SIM2*, *TBX3* and *TBX15*, respectively.

**Figure 2:**
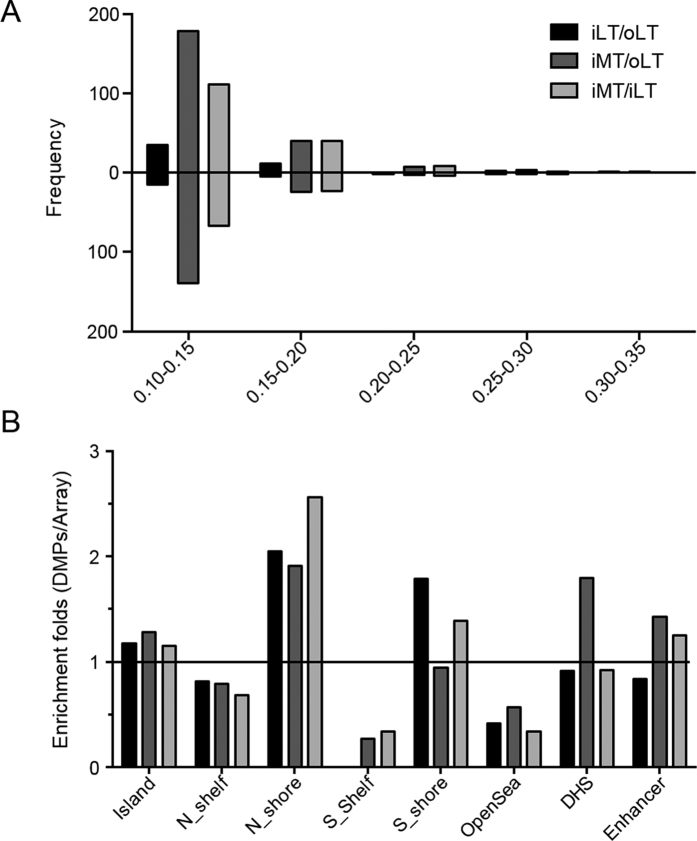
(**A**) Distribution of ∆β of the identified DMPs in the iLT/oLT, iMT/oLT and iMT/iLT groups. The bars above the X-axis indicate hypermethylation, the bars below the X-axis indicate hypomethylation. The Y-axis indicates the frequencies of hyper- and hypo-methylation in each range of |△β| values as indicated on the X-axis. (**B**) Enrichment folds of Genome features of identified DMPs compared to the Array probes in iLT/oLT, iMT/oLT and iMT/iLT groups. The X-axis: genome features, the Y-axis: the enrichment folds of the identified DMPs compared to the array probes. >1 indicates enrichment and <1 indicates diminishment. Island: >200 bp with GC percentage >50% and observed/expected CpG ratio >60%. Shore: up to 2 kb from CpG island. Shelf: 2–4 kb from CpG island. OpenSea: isolated CpGs in the genome. DHS: DNase I hypersensitive sites.

Three comparison groups, iLT vs oLT (iLT/oLT), iMT vs oLT (iMT/oLT), and iMT vs iLT (iMT/iLT) were analyzed to identify the DMPs associated with the intermediate stage, the late stage and the transition from the intermediate stage to the late stage of OA. In the iLT/oLT group, 72 significant DMPs covering 19 genes were identified, of which 22 (30.5%) DMPs were hypomethylated and 50 (69.5%) DMPs were hypermethylated. In the iMT/oLT group, 397 significant DMPs covering 135 genes were identified, which included 166 (41.8%) hypomethylated DMPs and 231 (58.2%) hypermethylated DMPs. In the iMT/iLT group, 257 DMPs covering 41 DMGs were identified, 95 (40%) DMPs were hypomethylated and 162 (60%) were hypermethylated. Heat map of the identified DMPs and β value plots of selected CpG examples from each group were shown in Fig. 1A,B. The top 20 DMPs with the most β value changes in the 3 comparison groups were listed in Tables 1, 2, 3. All the identified DMPs were listed in supplementary Table 1. Over half of the identified DMPs were hypermethylated and majority of them had |△β| of 10~20% in all three comparison groups. Only a very small percentage of DMPs (6.9% in iLT/oLT, 3.8% in iMT/oLT, and 1.9% in iMT/iLT) had |△β| of >20% (Fig. 2A).

### Genome features of identified DMPs

DNA methylation can exist in different patterns, such as CpG island, which is usually more than 200 bp containing >50% GC and observed/expected CpG ratio >60%. A shore is up to 2 kb from CpG island, a shelf is 2–4 kb from CpG island and opensea are isolated CpGs in the genome. DNA methylation sites with different genome features can play different roles in gene expression^13^. Thus, the genome features of the identified DMPs were first analyzed to identify the potential regulatory effect of methylation on gene expression. The three comparison groups showed consistent enrichment in the Island and N Shore, and diminishment in the Shelf and OpenSea (Fig. 2B). However, only iMT/oLT showed enrichment in the DHS (DNase I hypersensitive sites) region. Both iMT/oLT and iMT/iLT also showed enrichment in the enhancer region, (Fig. 2B). Methylation in DHS and enhancers often leads to altered gene expression, therefore, the different enrichment levels of DMPs in the three comparison groups may imply the diverse regulation of methylation in different stages.

### Gene Ontology and predicated biological functions

To systematically extract biological function annotations from the identified DMGs, DAVID tools were then used to study the gene ontology and functional categories. 19 DMGs identified in iLT/oLT were significantly enriched in developmental protein category (Benjamini-p = 0.001969, enrichment fold =9.6). A total of 122 DMGs from iMT/oLT were analyzed and were found significantly enriched in developmental pattern formation and specification, the skeletal system morphogenesis and development, DNA binding and transcription factor activity, metabolic process of nucleoside and nitrogen compound (Supplementary Table 2). Similarly, the 41 DMGs in iMT/iLT group were enriched in similar pathways (Supplementary Table 2), suggesting regulation on gene transcription and bone remodeling process.

Many of the identified DMGs were found to associate with OA or bone remodeling. For example, COL4A1, a structural extracellular molecule, has been shown to be differentially expressed in the subchondral bone of hip OA^14^. *IFRD1*, hypermethylated in iLT/oLT, was identified by GWAS to associate with hip OA^15^. IFRD1 could suppress bone formation and promote bone resorption through modulating the NF-κB/Smad/Osx and β-catenin/OPG pathways^16^, showing a pivotal role in bone remodeling and homeostasis. FAM5C, a soluble osteoblast differentiation factor which could maintain the phenotype and promote the differentiation of osteoblasts^17^. NPR3, a receptor of C-type natriuretic peptide (CNP), is a clearance inactivation receptor of CNP thus negatively regulate the NPR2 signaling, modulate the homeostasis of both cartilage and subchondral bone^18^. DNER was found to be upregulated in the affected knee OA cartilage^11^,^19^. Novel DMGs associated with OA progression were also identified in our study. For example, *WNT11*, hypermethylated in iLT/oLT, was a ligand for non-canonical Wnt/PCR pathway and had a critical role in osteogenesis^20^, which may also play a role in bone remodeling of OA.

### Expression of DMGs and correlation to methylation levels

**Figure 3:**
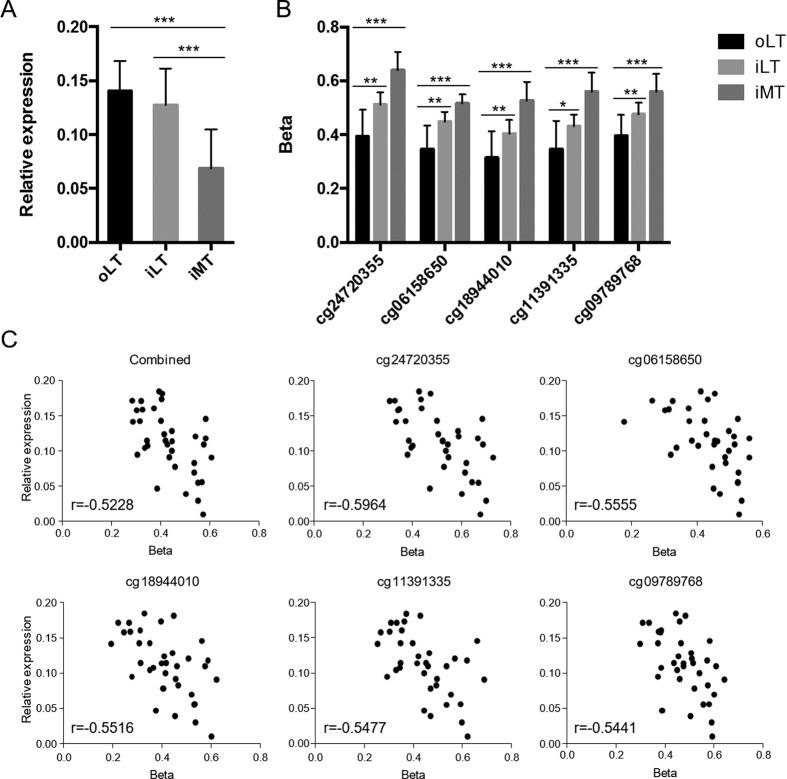
Hypermethylation in iLT and iMT regions is associated with a decrease in expression of *TBX15*. (**A**) The relative expression of *TBX15* in subchondral bone of the oLT, iLT and iMT regions. (**B**) Beta values of the top 5 CpGs that showed the most correlation to the *TBX15* expression in the subchondral bone of oLT, iLT and iMT regions. (**C**) Scatter plot showing the correlation between gene expression and the methylation for *TBX15*. The plot showing correlation for all the 26 CpGs combined and the top 5 CpGs are illustrated. r is the Spearman’s rank coefficient. Supplementary Table 5 listed the r and p value for all the correlated CpGs. p<: *, 0.05; **, 0.01; ***, 0.001.

A cluster of transcription regulators was identified among the DMGs. Specifically, of the 4 transcription regulators found in iLT/oLT, 33 in iMT/oLT and 20 in iMT/iLT, 2, 19 and 13 belong to the homeobox gene family, respectively (Supplementary Table 3). Some of the DMGs included multiple DMPs (Supplementary Table 4). For example, *EVX1* in iLT/oLT had 4 DMPs in 5′UTR, TSS1500 and 1^st^ exon. In iMT/oLT, *TBX15* had 26 DMPs in 5′UTR, TSS1500 and 1^st^ exon, and *HOXB3* had 20 DMPs in the 5′UTR, 1^st^ exon and gene body. This indicated methylation may play an important role on transcription in these genes. To evaluate this hypothesis, DMGs with large methylation alterations and multiple DMPs, which increase the possibility of methylation regulation, were selected to examine the expression level. *EVX1*, *PTPRN2*, *TBX3*, *SHOX2* and *TBX15* were selected. *TBX15* exhibited significant down regulation in both the iLT and the iMT in the subchondral bone (Fig. 3A). In order to investigate the correlation between expression and methylation, Spearman’s test was calculated for each of the identified DMPs associated with *TBX15*. Significant negative correlation was observed for all the DMPs, and the correlation for combined DMPs was also significant, with spearman’s r of −0.5228 (Supplementary Table 5). The methylation β values of the five DMPs showing highest correlation in the three regions were shown in Fig. 3B, and the correlation plots were illustrated in Fig. 3C. *EVX1* was found to be significantly downregulated only in the iMT region, but no correlation was identified for any of the associated DMPs (Supplementary Figure 3). The expression of *PTPRN2*, *TBX3* and *SHOX2* showed no significant difference across the three regions.

A combined analysis using our previous iMT/oLT expression array dataset^10^ was conducted to identify genes showing both methylation and expression changes (patient samples were different from those used in the current study). 11 genes showed both methylation and expression changes (Supplementary Table 6). However, from our observations, no consistent methylation~expression pattern could be identified, indicating other mechanisms of gene expression regulation in OA subchondral bone.

### Ingenuity pathway analysis of DMGs in iLT/oLT, iMT/oLT and iMT/iLT

**Figure 4:**
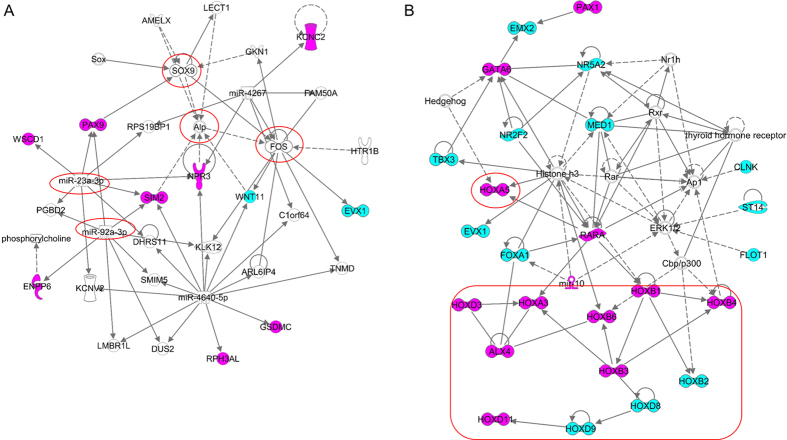
Predicted top networks associated with DMGs identified in iLT/oLT (**A**) and iMT/oLT (**B**) by IPA. Pink: hyper-methylated, blue: hypo-methylated. (**A**) The top network in iLT/oLT of subchondral bone showed several centered nodes in the red circle, SOX9, Alp, FOS, miR-23a and miR-92a were known in bone remodeling. (**B**) Network B indicates the enrichment of homeobox genes (in the rounded rectangle).

To comprehensively explore the underlying biological function associated with DMGs during OA progression in subchondral bone, IPA was employed to identify the enriched canonical pathways, up-stream regulators, and associated networks. The top canonical pathways enriched in the iLT/oLT DMGs were cAMP-mediated signaling (P = 1.58 × 10^−2^), G-protein coupled receptor signaling (P = 2.18 × 10^−2^) and tRNA splicing (P = 3.59 × 10^−2^), which were the same pathways observed in the iMT/oLT region of cartilage^12^, suggesting the methylation changed earlier in the subchondral bone than the cartilage. The upstream regulators identified in iLT/oLT included miR-4503 (P = 6.52 × 10^−4^), EPHB4 (P = 6.60 × 10^−4^), RIPPLY3 (P = 7.78 × 10^−4^), miR-642a-5p (P = 1.10 × 10^−3^) and miR-3655 (P = 1.54 × 10^−3^). EPHB4 signaling was shown to participate in osteogenesis and could protect subchondral bone and cartilage during OA with its ligand ephrin-B2^21^,^22^,^23^. The top network identified in iLT/oLT DMGs was illustrated in Fig. 4A. Five nodes (SOX9, FOS, Alp, miR-23a-3p and miR-92a-3p) were found to be centered on, which have been demonstrated important in bone development and metabolism^24^,^25^,^26^,^27^,^28^,^29^,^30^,^31^.

Three top canonical pathways were identified in iMT/oLT: the role of Oct4 in mammalian embryonic stem cell pluripotency (P = 8.09 × 10^−6^), transcription regulatory network in embryonic stem cells (P = 1.77 × 10^−3^) and tRNA splicing (P = 1.87 × 10^−2^), implying the probable reparation involving stem cells. Top upstream regulators were SF3B2 (P = 1.01 × 10^−10^), HOXB1 (P = 1.25 × 10^−8^), EED (P = 9.22 × 10^−8^), SHH (P = 2.77 × 10^−2^) and RNF2 (P = 4.12 × 10^−2^). SHH (Sonic Hedgehog) has a critical role in the chondrogenesis and osteogenesis^32^,^33^,^34^,^35^. RNF2 is a member of the polycomb family, which are chromatin modifiers responsible for mediating the epigenetic silencing of target genes. RNF2 was critical for neural crest-derived cartilage precursors to differentiate into chondrocytes^36^. The top associated network presented a cluster of transcription factors from homeobox gene family (Fig. 4B), which is consistent to the findings of methylation study on site-matched iMT/oLT OA cartilage^12^.

The top two canonical pathways identified in iMT/iLT are: transcriptional regulatory network in embryonic stem cells (P = 5.14 × 10^−5^) and role of Oct4 in mammalian embryonic stem cell pluripotency (P = 3.13 × 10^−3^). Top upstream regulators are KAT6A, HOXB1, SF3B2, NANOG and SOX2, all of which are involved in either the development or RNA splicing. The top associated network presented a clustered of HOX family and centered on histone H3 (Supplementary Figure 4A).

### DMGs and pathways shared between OA subchondral bone and cartilage

To comprehensively understand the interactions between subchondral bone and cartilage during OA progression, DMPs and DMGs identified in the subchondral bone and cartilage were compared. As no DMPs were identified in the iLT/oLT of cartilage, thus only the DMPs and DMGs in the iMT/oLT groups were compared. The iMT/oLT groups of cartilage and subchondral bone shared 111 DMPs (supplementary Table 7) and 41 DMGs (Table 4). The 111 DMPs showed high consistency in methylation alterations (supplementary Table 7), signifying the common alterations at the late stage of OA in both the subchondral bone and cartilage.

GO analysis of the 41 shared DMGs revealed higher enrichment folds in the morphogenesis and development of skeletal system than subchondral bone or cartilage alone (Supplementary Table 2). Networks associated with the shared DMGs identified by IPA also revealed a cluster of homeobox transcription factors (Supplementary Figure 4B), and centered on the TGFB node (Supplementary Figure 4C), which was well known for the significance of TGF-β signaling in cartilage homeostasis and subchondral bone remodeling. Top canonical pathways associated with the shared DMGs were tRNA splicing (1.91 × 10^−3^) and role of Oct4 in mammalian embryonic stem cell pluripotency (3.19 × 10^−3^). These features together pointed towards the reparation involved stem cells at the late stage of OA both in subchondral bone and cartilage.

## Discussion

In this study, we adopted a unique three-region (oLT, iLT and iMT) model system on the tibial plateau of knee joint to represent the early, intermediate and late stages of OA^9^,^11^, and performed three comparisons (iLT/oLT, iMT/oLT and iMT/iLT) to identify methylation alterations associated with the intermediate, late and the transition from intermediate to late stage of OA. Significant DMPs and DMGs were identified in all the three groups. DMGs that were found to associate with OA in previous studies were also identified in our study, such as, *COL4A1*, *IFRD1* and *DNER*. DNER, which was found to be up-regulated in the damaged region of cartilage in knee OA^11^,^19^, was found to be hypomethylated in iMT/iLT group in this study. This suggested that DNER also played a role during the transition from intermediate stage to the late stage of OA in the subchondral bone.

A cluster of transcription factors was identified and many of them showed multiple methylation alterations sites (Supplementary Tables 3 and 4), suggesting the regulation of methylation on gene expression. In our study, expression of *TBX15* was found to be significantly downregulated during OA progression, and negatively correlated to the methylation level of the associated 26 DMPs (Fig. 3 and supplementary Table 5). *TBX15* was hypermethylated at the promoter region (TSS1500, TSS200, 5′UTR and 1^st^ exon), which is concordant with that the hypermethylation in promotor region of a gene usually associate with expression down regulation. TBX15 is critical in the development of bone and cartilage through regulating mesenchymal precursor cells and prehypertrophic chondrocytes^37^. A combined analysis with previous expression array data in subchondral bone at the late stage of OA identified 11 overlapped genes showing both methylation and expression alterations but no consistent methylation~expression pattern could be identified. However, the methylation~expression pattern was identified in the cartilage from our previous study^12^. This may due to the sole cell type in cartilage and multiple cell types in subchondral bone, and other mechanisms that regulate gene expression, for instance, microRNAs.

Function network identified in the iLT/oLT group showed several centered nodes that featured in the pathology of OA (Fig. 4A). ALP is a main osteogenesis index which is increased when active bone formation occurs. SOX9 is a key transcription factor in normal skeletal development, especially in chondrogenesis^24^. FOS has a critical function in bone development due to its regulatory effects on all types of bone cells^25^,^26^. MiR-23 and miR-92a could regulate osteoblast differentiation through targeting on bone-specific transcription factor RUNX2^27^,^29^,^30^,^31^. The associated networks identified in iMT/oLT highlighted a cluster of HOX transcription factors (Fig. 4B), together with the top canonical pathways that the role of Oct4 in mammalian embryonic stem cell pluripotency and the transcription regulatory network in embryonic stem cells, implying the potential tissue reparation.

One merit of our study was the opportunity to compare cartilage with the site-matched subchondral bone. The DMPs identified in the cartilage and subchondral bone showed different methylation pattern and genome features, suggesting the tissue-specific role of methylation during disease. No DMPs were identified in the iLT/oLT of cartilage, while 72 DMPs were identified in the underlying subchondral bone. In addition, canonical pathways identified in the subchondral bone at the intermediate stage were exactly the same pathways identified in cartilage at the late stage of OA. These observations suggest that changes in subchondral bone may precede changes in the cartilage. At the late stage, the subchondral bone and cartilage shared 111 DMPs and 41 DMGs, suggesting coordination of common pathways and mechanisms were employed in both tissue. GO analysis of the 41 shared DMGs did show much higher enrichment in the morphogenesis and development of skeletal system. Networks associated with the shared DMGs highlighted TGF-β1 signaling and a shared cluster of HOX transcription factors (Supplementary Figure 4B,C), implying the common pathological pathway of TGF-β1 and the potential tissue reparation.

Accumulating evidence suggested there are interactions between subchondral bone and cartilage during OA progression. The vascularization, bone marrow lesions and micro-cracks in subchondral bone and cartilage could facilitate the interchange of molecules. Among DMGs identified in subchondral bone, *EFEMP1*, encoding an extracellular matrix protein named fibulin-3, could inhibit the chondrocyte differentiation in cartilage^38^. *IGFBP5*, encoding an insulin-like growth factor binding protein, was up-regulated in both subchondral bone and cartilage in OA^39^,^40^, and could promote chondrocyte differentiation through enhancement of the IGF-I effect^41^. Among the DMGs identified in OA cartilage, *BMP6* and *GREM2* could function on osteoblasts in the subchondral bone. BMP6 was required for mesenchymal stem cells to differentiate into osteoblast, and it is also released by osteoclasts as a key bone coupling factor recruiting osteoblasts to the resorption site, thus is a critical regulator in bone remodeling^42^. GREM2 was a negative regulator of BMP function during osteoblast differentiation^43^. A GWAS study found a genetic variant in the *FMN2/GREM2* locus influences GREM2 expression in osteoblasts and thereby trabecular number and thickness^44^. In addition, DMGs involved in angiogenesis were identified. miR-10a was found to promote angiogenesis. Overexpression of miR-10a *in vitro* could increase the VEGF expression in mouse osteogenic cells and promoted tube formation in mouse vein endothelial cells^45^. These observations suggested that the methylation is one mechanism to regulate the molecular crosstalk between subchondral bone and cartilage in OA (Supplementary Figure 5).

Two DNA methylation studies conducted on bone tissue of OA have been reported. One early study examined the methylome of trabecular bone from hip OA compared to hip fracture and identified 45 CpGs with differential methylation >0.1, which had much less CpGs than ours. It might be caused by using Infinium HumanMethylation27 BeadChips, which included much less probes than the updated Infinium HumanMethylation450 BeadChips used in this study. No overlapped genes were found, although the HOX transcription factors and pathways of development of skeletal pathway were also identified. This might due to the different study design and different tissue used from our study. The study compared the methylome of trabecular bone from hip OA and hip fracture, but not subchondral bone underlying intact cartilage and eroded cartilage of hip OA^7^. Our study design is very similar to that of the recent second study except that we included the intermediate stage of the disease and the tissue is knee but not hip. The recent study identified 7316 DMPs covering 2279 DMGs, with majority hypomethylated, which was opposite to our findings^8^. IPA of DMGs identified in hip OA highlighted the inflammatory pathways and upstream regulators, suggesting the key role of inflammation in hip OA^8^, which was consistent to the findings from cartilage of hip OA^46^. However, in our study of knee OA, the HOX genes were found most enriched by IPA. Sixty-four DMGs were identified same in our study. GO analysis showed they were most enriched in DNA binding region: homeobox. Both our study and the recent study showed common pathways were shared in the cartilage and subchondral bone^8^. Our and others’ methylation studies of cartilage and subchongdral bone indicated the different etiologies of hip OA and knee OA^8^,^46^,^47^.

Our study has several limitations. The sample size used in this study was small, and non-OA knee joint tissues were not available as control due to difficulties of collecting these tissues. From our previous expression study using the same model system, cartilage from the same three regions (oLT, iMT and iLT) on non-OA joints clustered together next to the oLT region of the OA joints^11^. Thus, oLT could serve as an alternative normal control which could reduce the inter-individual variations and reduce the limitations caused by small sample size. Additional cohort was not available for replicating the top DMG identified from this study. Lastly, although we compared the methylomes of subchondral bone and cartilage, and identified the common pathways, we were not able to determine the exact interactions between the subchondral bone and cartilage. Further analysis will be required to explore the interactions between the two layers of tissues.

In conclusion, our study not only identified DNA methylation alterations associated with knee OA progression in the subchondral bone, but also compared the results with that of the site-matched cartilage. Our results provided evidence that changes in the subchondral bone could precede the methylation changes in the cartilage. DMPs showed different genome features in the subchondral bone and cartilage and DMGs were enriched in the development and morphogenesis of the skeletal system. The enrichment in HOX genes and Oct4 pathways implied the potential tissue reparation at the late stage of OA. Analysis of shared DMGs in subchondral bone and cartilage reinforced this speculation. Methylation is one mechanism that regulates the crosstalk between cartilage and subchondral bone. Together, our study contributes to the understanding of DNA methylation in the subchondral bone during OA progression, and shed light on the future therapy through tissue reparation.

## Methods

### Knee joint tissues

The 12 knee joints used in this study were isolated from patients who underwent joint replacement surgery due to primary OA and were also used in our previous methylation study on the cartilage^12^. Patient demographics are shown in supplementary Table 8. The diagnosis of OA was based on the criteria of the American College of Rheumatology. All the tibial plateaus had the same compartment pattern of cartilage erosion and all the knees were medially involved in the disease (Supplementary Figure 1). Informed consent was obtained from each patient enrolled in this study. This study was approved by all the participating institutions (Sagamihara National Hospital, University of Tokyo and RIKEN) with the approval No. Yokohama H17-23(6). The methods were carried out in accordance with the Declaration of Helsinki.

### Subchondral bone sampling and DNA/RNA extraction

The three regions on the tibial plateaus were sectioned using homemade cutting station described previously (Supplementary Figure 1B)^9^. The subchondral bone powder was collected by a high-speed rotator after obtaining the site-matched cartilage. The entire processing was conducted in the liquid nitrogen to maintain the original disease state and to prevent shear damage^9^. In our system, subchondral bone refers to the total subarticular tissue under the calcified cartilage. This includes the subchondral bone plate and subarticular spongiosa and extends to approximately 2–5 mm depth. Our previous work showed that these regions could encompass a full range of histological severity in knee OA cartilage and changes in subchondral bone highly correlated with cartilage degradation^9^.

Genomic DNA was then extracted from about 100 mg of subchondral bone powder using QIAamp DNA Mini Kit (QIAGEN, Tokyo, Japan). RNA was extracted according to the established protocol^9^. DNA and RNA were isolated from adjacent bone fragments from the same area.

### Methylation profiling

After examining the quality and quantity by agarose gel electrophoresis Spectrophotometer (SpectraMax Plate Reader, Molecular Devices, Sunnyvale, CA), DNA (2 μg) was bisulfite treated using EZ DNA methylation kit (Zymo Research, Irvine, CA), and methylation profile was then assessed by Infinium HumanMethylation450 BeadChips (Illumina, San Diego, CA, USA).

### Data processing and statistical analysis

Raw data was read into R using the Bioconductor *minfi* package (R version 3.2.2; http://www.r-project.org/). Principle component analysis (PCA) was performed in R to identify outliers, and probe filtering and normalization was performed by built-in function of *minfi* package to exclude the non-eligible probes (detection p-value > 0.01 in more than 5% samples, on X/Y chromosomes and 65 SNP probes on Illumina manifest). Beta values were used to express the methylation level, and M values, the logarithmic transform of β values, were used in the statistical analysis which adopted F test to identify DMPs in the iLT/oLT and iMT/oLT comparison groups. Probes with FDR-corrected p value < 0.05 and mean |△β| > 0.1 were considered significant. Heat maps of identified DMPs were generated in R.

Paired student’s t test was used in comparisons of the relative expression and β values of the selected individual DMPs. Spearman’s rank correlation coefficient was used to evaluate the correlation between the expression and the methylation of each CpG.

### cDNA synthesis and real-time PCR

cDNA library was constructed using Superscript III First-Strand Synthesis SuperMix (ThermoFisher Scientific, Tokyo, Japan). Real-time PCR was conducted using SYBR Premix Ex Taq II (TAKARA, Tokyo, Japan) on Applied Biosystems 7900HT. Relative expression of interested genes to *GAPDH* was calculated using 2^−ΔCt^ method. Primers for real-time PCR were listed in Supplementary Table 9.

### Bisulfite Sanger sequencing

To verify the methylation chip data, probes in *HOXA3*, *HOXA5* and *HOXA9* were selected based on the bimodal distribution of β values, and their methylation levels were determined using bisulfite Sanger sequencing reported previously^12^. The same DNA samples used in the methylation chip were used in bisulfite Sanger sequencing.

### Gene ontology

DAVID tools (https://david.ncifcrf.gov/home.jsp) was used to perform gene ontology (GO) analysis^48^. Briefly, the identified DMGs were converted by Gene Accession Conversion Tool and then submitted to Functional Annotation. All 19 DMGs in iLT/oLT group, 41 DMGs in iMT/iLT group of subchondral bone and 41 shared DMGs in iMT/oLT of subchondral bone and cartilage were successfully converted and analyzed. 122 DMGs in iMT/oLT of subchondral bone were successfully converted and analyzed. Default settings of Functional_Categories (COG_Ontology, SP_PIR_Keywords, UP_SEQ_Feature) and Gene_Ontology (GOTERM_BP_FAT, GOTERM_CC_FAT, GOTERM_MF_FAT) was used for subsequent analysis. Fold enrichment was used as the enrichment index. Benjamini-corrected p-value < 0.05 was considered significant.

### Ingenuity pathway analysis

Ingenuity Pathway Analysis (IPA, QIAGEN Redwood City, www.qiagen.com/ingenuity) was used to identify enriched canonical pathways, predict potential biological interaction networks and the upstream regulators from the identified DMGs under the default settings. All 19 DMGs identified in oLT/iLT and 130 DMGs in oLT/iMT were performed IPA. 5 DMGs in iMT/oLT group and 2 DMGs in iMT/iLT group were not included in the IPA database thus were not analyzed by IPA. 41 shared DMGs in iMT/oLT of subchondral bone and cartilage were also analyzed by IPA.

## Additional Information

**How to cite this article**: Zhang, Y. *et al*. Identification of DNA methylation changes associated with disease progression in subchondral bone with site-matched cartilage in knee osteoarthritis. *Sci. Rep.* **6**, 34460; doi: 10.1038/srep34460 (2016).

## Supplementary Material

Supplementary Information

## Figures and Tables

**Table 1:** Top 20 differentially methylated CpGs in iLT/oLT subchondral bone.

Illumina ID	Associated gene	oLT mean β (SD)	iLT mean β (SD)	Mean △β	FDR-p value
Hypomethylated					
cg05209996	/	0.6360 (0.0861)	0.3402 (0.0944)	−0.2958	1.66E-03
cg05401764	*SHOX2*	0.7353 (0.0424)	0.4881 (0.1188)	−0.2471	4.44E-03
cg07104209	*EVX1*	0.6163 (0.0322)	0.4406 (0.0457)	−0.1757	1.89E-05
cg05825073	*EVX1*	0.4709 (0.0554)	0.3021 (0.0444)	−0.1689	9.25E-04
cg27397850	*EVX1*	0.5680 (0.0456)	0.4041 (0.0492)	−0.1639	9.25E-04
cg03458172	*SHOX2*	0.6258 (0.0468)	0.4718 (0.0603)	−0.1540	4.44E-03
cg25506747	*SHOX2*	0.6298 (0.0458)	0.4781 (0.0633)	−0.1518	5.66E-03
cg21437028	*SHOX2*	0.6756 (0.0455)	0.5359 (0.0407)	−0.1396	1.51E-03
cg19818890	*/*	0.4956 (0.0435)	0.3573 (0.0523)	−0.1383	4.39E-03
cg21552242	*SHOX2*	0.7862 (0.0350)	0.6521 (0.0500)	−0.1341	2.29E-03
cg10445315	*/*	0.7612 (0.0329)	0.6456 (0.0510)	−0.1156	7.30E-03
cg08787251	*EFEMP1*	0.7194 (0.0231)	0.6070 (0.0443)	−0.1124	1.43E-03
cg02579136	*WNT11*	0.4300 (0.0127)	0.3200 (0.0647)	−0.1100	3.44E-02
cg06496728	*/*	0.5736 (0.0440)	0.4644 (0.0484)	−0.1092	1.83E-02
cg10526277	*/*	0.5134 (0.0331)	0.4044 (0.0542)	−0.1091	1.62E-02
cg15726154	*SHOX2*	0.7314 (0.0332)	0.6227 (0.0579)	−0.1087	2.31E-02
cg08654915	*/*	0.6795 (0.0337)	0.5710 (0.0454)	−0.1085	6.69E-03
cg24028202	*ISLR2*	0.7138 (0.0265)	0.6053 (0.0274)	−0.1085	8.42E-05
cg21847036	*SHOX2*	0.6747 (0.0338)	0.5691 (0.0418)	−0.1056	5.41E-03
cg19575372	*/*	0.5112 (0.0444)	0.4062 (0.0369)	−0.1050	9.84E-03
Hypermethylated					
cg12659494	*SIM2*	0.4680 (0.0529)	0.6015 (0.0305)	0.1335	1.94E-03
cg23971987	*/*	0.4716 (0.0437)	0.6078 (0.0503)	0.1362	3.91E-03
cg00011482	*PAX9*	0.4093 (0.0530)	0.5460 (0.0283)	0.1367	1.53E-03
cg00616687	*SIM2*	0.1700 (0.0492)	0.3134 (0.0831)	0.1434	3.38E-02
cg19241327	*PTPRN2*	0.3226 (0.0725)	0.4708 (0.0278)	0.1483	1.08E-02
cg10720723	*PTPRN2*	0.3419 (0.0511)	0.4922 (0.0474)	0.1503	2.51E-03
cg26295921	*PTPRN2*	0.4138 (0.0680)	0.5642 (0.0447)	0.1504	9.87E-03
cg04415798	*PAX9*	0.5375 (0.0641)	0.6898 (0.0424)	0.1523	4.75E-03
cg25977493	*SHOX2*	0.2517 (0.0495)	0.4050 (0.0602)	0.1533	6.07E-03
cg22571664	*NPR3*	0.2352 (0.0516)	0.3971 (0.0644)	0.1620	6.38E-03
cg15634747	*/*	0.4689 (0.0582)	0.6309 (0.0602)	0.1621	6.04E-03
cg00266286	*NPR3*	0.2563 (0.0766)	0.4196 (0.0759)	0.1633	3.44E-02
cg23683588	*PAX9*	0.4778 (0.0703)	0.6485 (0.0588)	0.1707	8.15E-03
cg21207665	*PAX9*	0.4385 (0.0482)	0.6126 (0.0711)	0.1741	5.66E-03
cg15572489	*PTPRN2*	0.3275 (0.0630)	0.5116 (0.0646)	0.1841	4.09E-03
cg04937416	*PTPRN2*	0.3379 (0.0744)	0.5263 (0.0741)	0.1884	1.14E-02
cg22976224	*SIM2*	0.3910 (0.0778)	0.5861 (0.0744)	0.1951	1.31E-02
cg08297082	*SIM2*	0.3338 (0.0619)	0.6053 (0.0333)	0.2715	2.52E-06
cg27325152	*SIM2*	0.3166 (0.0771)	0.6101 (0.0427)	0.2935	1.87E-05
cg03993743	*SIM2*	0.2909 (0.0594)	0.6249 (0.0637)	0.3339	2.52E-06

**Table 2:** Top 20 differentially methylated CpGs in iMT/oLT subchondral bone.

Illumina ID	Associated gene	oLT mean β (SD)	iMT mean β (SD)	Mean △β	FDR-p value
Hypomethylated					
cg19346371	*TBX3*	0.5096 (0.1013)	0.2397 (0.0754)	−0.2699	1.957E-03
cg04693928	*LMX1B*	0.6048 (0.0915)	0.3749 (0.1143)	−0.2299	9.579E-03
cg15526081	*/*	0.6488 (0.0802)	0.4256 (0.1062)	−0.2232	6.207E-03
cg08384314	*IER3;FLOT1*	0.5505 (0.0693)	0.3509 (0.0795)	−0.1996	4.161E-03
cg13630043	*EMX2*	0.6548 (0.0610)	0.4670 (0.0842)	−0.1878	4.099E-03
cg04368796	*NKIRAS2*	0.6295 (0.0915)	0.4491 (0.0806)	−0.1804	1.187E-02
cg06141846	*EMX2OS*	0.5556 (0.0727)	0.3833 (0.0725)	−0.1723	6.647E-03
cg17147211	*/*	0.6678 (0.0603)	0.4992 (0.1410)	−0.1686	4.669E-02
cg16489193	*VPS52; RPS18*	0.5554 (0.0536)	0.3874 (0.0932)	−0.1679	9.786E-03
cg11375458	*/*	0.6331 (0.0789)	0.4663 (0.0775)	−0.1668	1.081E-02
cg22920873	*C7orf55*	0.3152 (0.0675)	0.1488 (0.0429)	−0.1663	1.907E-03
cg18561589	*EMX2OS*	0.6211 (0.0708)	0.4553 (0.0843)	−0.1658	1.050E-02
cg27630311	*TBX3*	0.4184 (0.1064)	0.2532 (0.0717)	−0.1652	2.386E-02
cg25556690	*/*	0.4841 (0.1029)	0.3191 (0.0902)	−0.1650	3.397E-02
cg08406102	*EMX2OS*	0.4327 (0.0651)	0.2704 (0.0581)	−0.1623	4.185E-03
cg08451832	*/*	0.4677 (0.0905)	0.3068 (0.0737)	−0.1609	1.943E-02
cg03311684	*EMX2OS*	0.6464 (0.0783)	0.4878 (0.0648)	−0.1586	8.787E-03
cg21472506	*OTX1*	0.7089 (0.0571)	0.5504 (0.1136)	−0.1586	2.471E-02
cg23229261	*OTX1*	0.6422 (0.0724)	0.4860 (0.1139)	−0.1563	3.634E-02
cg24725789	*/*	0.6976 (0.0521)	0.5415 (0.0720)	−0.1561	4.346E-03
Hypermethylated					
cg19175386	*/*	0.4923 (0.0952)	0.6733 (0.0670)	0.1810	1.031E-02
cg05172122	*TBX15*	0.3232 (0.0725)	0.5059 (0.0755)	0.1826	5.143E-03
cg08942939	*TBX15*	0.3097 (0.0824)	0.4926 (0.0739)	0.1829	6.611E-03
cg23371746	*TBX15*	0.3829 (0.0824)	0.5660 (0.0578)	0.1830	4.442E-03
cg19730691	*TBX15*	0.4596 (0.0707)	0.6471 (0.0511)	0.1874	1.827E-03
cg03942051	*TBX15*	0.2408 (0.0833)	0.4343 (0.0634)	0.1935	3.366E-03
cg10967023	*/*	0.2632 (0.0961)	0.4568 (0.1006)	0.1936	1.331E-02
cg26272623	*TBX15*	0.2534 (0.0739)	0.4490 (0.0601)	0.1956	2.067E-03
cg26127662	*HOXA3*	0.5046 (0.0757)	0.7057 (0.0621)	0.2011	2.297E-03
cg22820316	*TBX15*	0.3460 (0.0868)	0.5481 (0.0629)	0.2020	3.391E-03
cg18944010	*TBX15*	0.3148 (0.0983)	0.5268 (0.0696)	0.2120	5.267E-03
cg11391335	*TBX15*	0.3465 (0.1043)	0.5604 (0.0697)	0.2139	5.839E-03
cg13655674	*TBX15*	0.2983 (0.1111)	0.5137 (0.0504)	0.2154	4.785E-03
cg24672833	*/*	0.2891 (0.1133)	0.5132 (0.1168)	0.2241	2.225E-02
cg24884142	*TBX15*	0.3697 (0.0892)	0.5974 (0.0543)	0.2277	1.827E-03
cg24720355	*TBX15*	0.3936 (0.0985)	0.6397 (0.0679)	0.2461	2.300E-03
cg10308785	*LOC404266; HOXB6*	0.3183 (0.1281)	0.5691 (0.0891)	0.2508	7.922E-03
cg16990168	*TBX15*	0.3482 (0.1203)	0.6260 (0.0746)	0.2778	2.899E-03
cg25340966	*TBX15*	0.3221 (0.1353)	0.6143 (0.0865)	0.2922	4.346E-03
cg10703826	*TBX15*	0.2995 (0.1342)	0.6268 (0.1081)	0.3273	3.370E-03

**Table 3:** Top 20 differentially methylated CpGs in iMT/iLT subchondral bone.

Illumina ID	Associated Gene	iLT mean β (SD)	iMT mean β (SD)	Mean △β	FDR-p value
Hypermethylated					
cg19346371	TBX3	0.5020 (0.1141)	0.2397 (0.0754)	−0.2622	4.91E-03
cg23229261	OTX1	0.7226 (0.0638)	0.4860 (0.1139)	−0.2367	5.50E-03
cg24039697	FLJ45983	0.5550 (0.0895)	0.3294 (0.0989)	−0.2256	1.14E-02
cg21472506	OTX1	0.7586 0.0516)	0.5504 (0.1136)	−0.2082	8.00E-03
cg15526081		0.6311 0.0721)	0.4256 (0.1062)	−0.2056	1.65E-02
cg04937416	PTPRN2	0.5263 0.0710)	0.3456 (0.0707)	−0.1807	8.74E-03
cg13630043	EMX2	0.6428 0.0582)	0.4670 (0.0842)	−0.1758	9.97E-03
cg09334277		0.6822 (0.0668)	0.5078 (0.0574)	−0.1744	4.18E-03
cg03301200		0.5306 (0.0367)	0.3564 (0.0852)	−0.1742	5.92E-03
cg02019574		0.5718 (0.0443)	0.3992 (0.1109)	−0.1726	3.96E-02
cg08451832		0.4794 (0.0875)	0.3068 (0.0737)	−0.1726	3.34E-02
cg03993743	SIM2	0.6249 (0.0610)	0.4567 (0.0983)	−0.1681	3.35E-02
cg18561589	EMX2OS	0.6227 (0.0584)	0.4553 (0.0843)	−0.1675	1.44E-02
cg06141846	EMX2OS	0.5493 (0.0725)	0.3833 (0.0725)	−0.1660	1.72E-02
cg03311684	EMX2OS	0.6492 (0.0639)	0.4878 (0.0648)	−0.1615	8.61E-03
cg27630311	TBX3	0.4143 (0.0827)	0.2532 (0.0717)	−0.1611	3.08E-02
cg21608600		0.5253 (0.0326)	0.3644 (0.0968)	−0.1610	1.81E-02
cg00756451	TBX5	0.4787 (0.0481)	0.3177 (0.0521)	−0.1609	1.60E-03
cg12121660	HOXB2	0.6276 (0.0443)	0.4698 (0.0799)	−0.1579	9.97E-03
cg15572489	PTPRN2	0.5116 (0.0619)	0.3555 (0.0719)	−0.1562	1.55E-02
Hypermethylated					
cg20307896		0.4289 (0.0567)	0.6071 (0.0588)	0.1781	1.97E-03
cg16856049		0.4274 (0.0643)	0.6078 (0.0777)	0.1804	9.49E-03
cg05259508		0.4211 (0.0779)	0.6021 (0.0442)	0.1810	3.99E-03
cg17616537	HOXB3	0.5390 (0.0562)	0.7214 (0.0748)	0.1825	3.66E-03
cg07080050	HOXC4	0.3924 (0.0555)	0.5762 (0.0369)	0.1838	3.96E-04
cg02458062	HOXB3	0.6429 (0.0534)	0.8307 (0.0855)	0.1878	4.02E-03
cg21229570		0.5882 (0.1032)	0.7760 (0.0359)	0.1878	9.06E-03
cg15772924	HOXC4; HOXC5; HOXC6	0.4023 (0.0505)	0.5923 (0.0373)	0.1900	1.95E-04
cg18470839		0.5661 (0.0661)	0.7574 (0.0383)	0.1913	5.75E-04
cg18220920		0.4180 (0.0794)	0.6126 (0.0520)	0.1945	3.60E-03
cg01529365		0.2868 (0.0619)	0.4882 (0.0684)	0.2014	2.11E-03
cg18197377		0.4571 (0.0871)	0.6607 (0.0588)	0.2036	5.66E-03
cg14283944		0.2543 (0.0540)	0.4588 (0.0848)	0.2045	2.52E-03
cg19175386		0.4625 (0.0830)	0.6733 (0.0670)	0.2107	4.38E-03
cg24208826		0.2459 (0.0884)	0.4590 (0.0846)	0.2131	1.16E-02
cg25340966	TBX15	0.3968 (0.0680)	0.6143 (0.0865)	0.2174	3.92E-03
cg10308785	HOXB6; LOC404266	0.3427 (0.1107)	0.5691 (0.0891)	0.2264	1.89E-02
cg26127662	HOXA3	0.4790 (0.0898)	0.7057 (0.0621)	0.2267	2.79E-03
cg24672833		0.2681 (0.0923)	0.5132 (0.1168)	0.2452	1.92E-02
cg10703826	TBX15	0.3675 (0.0649)	0.6268 (0.1081)	0.2593	2.98E-03

**Table 4:** Shared DMGs in iMT/oLT identified in subchondral bone and the site-matched cartilage.

Gene ID	Symbol	Entrez Gene Name
Gene ID	Symbol	Entrez Gene Name
*AGBL1*	AGBL1	ATP/GTP binding protein-like 1
*ALX4*	ALX4	ALX homeobox 4
*BAI3*	ADGRB3	adhesion G protein-coupled receptor B3
*C18orf2*	LINC00470	long intergenic non-protein coding RNA 470
*CLNK*	CLNK	cytokine-dependent hematopoietic cell linker
*COG2*	COG2	component of oligomeric golgi complex 2
*EMX2*	EMX2	empty spiracles homeobox 2
*EMX2OS*	EMX2OS	EMX2 opposite strand/antisense RNA
*ENPP6*	ENPP6	ectonucleotide pyrophosphatase/phosphodiesterase 6
*EXOC2*	EXOC2	exocyst complex component 2
*FAM124B*	FAM124B	family with sequence similarity 124B
*GDF6*	GDF6	growth differentiation factor 6
*GSDMC*	GSDMC	gasdermin C
*HOXA3*	HOXA3	homeobox A3
*HOXB1*	HOXB1	homeobox B1
*HOXB2*	HOXB2	homeobox B2
*HOXB3*	HOXB3	homeobox B3
*HOXD3*	HOXD3	homeobox D3
*HOXD8*	HOXD8	homeobox D8
*HOXD9*	HOXD9	homeobox D9
*IQSEC3*	IQSEC3	IQ motif and Sec7 domain 3
*IRX1*	IRX1	iroquois homeobox 1
*JAKMIP1*	JAKMIP1	janus kinase and microtubule interacting protein 1
*LMX1B*	LMX1B	LIM homeobox transcription factor 1, beta
*MBP*	MBP	myelin basic protein
*NR2F2*	NR2F2	nuclear receptor subfamily 2, group F, member 2
*PACRG*	PACRG	PARK2 co-regulated
*PAX9*	PAX9	paired box 9
*PCID2*	PCID2	PCI domain containing 2
*PDE4D*	PDE4D	phosphodiesterase 4D, cAMP-specific
*PEX5L*	PEX5L	peroxisomal biogenesis factor 5-like
*RPTOR*	RPTOR	regulatory associated protein of MTOR, complex 1
*SLC25A21*	SLC25A21	solute carrier family 25 (mitochondrial oxoadipate carrier), member 21
*LOC100129794*		
*TBX15*	TBX15	T-box 15
*TBX3*	TBX3	T-box 3
*TFAP2A*	TFAP2A	transcription factor AP-2 alpha (activating enhancer binding protein 2 alpha)
*TMEM67*	TMEM67	transmembrane protein 67
*TRERF1*	TRERF1	transcriptional regulating factor 1
*TRHR*	TRHR	thyrotropin-releasing hormone receptor
*USH2A*	USH2A	Usher syndrome 2A (autosomal recessive, mild)
