# Supplementary material for: Identification of DNA methylation changes associated with disease progression in subchondral bone with site-matched cartilage in knee osteoarthritis
Source: Sci Rep. 2016 Sep 30;6:34460. doi: 10.1038/srep34460 (PMC5043275; doi:10.1038/srep34460)
Supplement: Supplementary Information [file srep34460-s1.pdf]

Supplementary information

Identification of DNA methylation changes associated with disease progression in subchondral bone with site-matched cartilage in knee osteoarthritis

Yanfei Zhang Ph.D.[1, 9], Naoshi Fukui M.D., Ph.D.[2,3], Mitsunori Yahata M.Sc.[1,4], Yozo Katsuragawa M.D., Ph.D.[5], Toshiyuki Tashiro M.D.[6], Shiro Ikegawa M.D., Ph.D.[7], Ming Ta Michael Lee Ph.D.[1,8,9*]

1. Laboratory for International Alliance on Genomic Research, Center for Integrative Medical Sciences, RIKEN, Yokohama, Japan

2. Clinical Research Center, National Hospital Organization Sagamihara Hospital, Kanagawa, Japan

3. Department of Life Sciences, Graduate School of Arts and Sciences, the University of Tokyo, Tokyo, Japan

4. Laboratory for Pharmacogenomics, Center for Integrative Medical Sciences, RIKEN, Yokohama, Japan

5. Department of Orthopaedic Surgery, Center Hospital of the National Center for Global Health and Medicine Center Hospital, Tokyo, Japan

6. Department of Orthopaedic Surgery, Tokyo Yamate Medical Center, Tokyo, Japan

7. Laboratory for Bone and Joint Diseases, Center for Integrative Medical Sciences, RIKEN, Tokyo, Japan

8. Institute of Biomedical Sciences, Academia Sinica, Taipei, Taiwan

9. Genomic Medicine Institute, Geisinger Health System, Danville, PA, USA

* Address correspondence to Dr. Ming Ta Michael Lee, Genomic Medicine Institute, Geisinger Health System, Danville, PA17822, USA. Email: mlee2@geisinger.edu; mingta@gmail.com

Supplementary table 1: All DMPs identified in iLT/oLT and iMT/oLT in subchondral bone

Illumina ID	Associated Gene	Mean ∆β	FDR-p value
iLT/oLT			
cg03993743	SIM2	0.3339	2.52E-06
cg27325152	SIM2	0.2935	1.87E-05
cg08297082	SIM2	0.2715	2.52E-06
cg22976224	SIM2	0.1951	1.31E-02
cg04937416	PTPRN2	0.1884	1.14E-02
cg15572489	PTPRN2	0.1841	4.09E-03
cg21207665	PAX9	0.1741	5.66E-03
cg23683588	PAX9	0.1707	8.15E-03
cg00266286	NPR3	0.1633	3.44E-02
cg15634747		0.1621	6.04E-03
cg22571664	NPR3	0.1620	6.38E-03
cg25977493	SHOX2	0.1533	6.07E-03
cg04415798	PAX9	0.1523	4.75E-03
cg26295921	PTPRN2	0.1504	9.87E-03
cg10720723	PTPRN2	0.1503	2.51E-03
cg19241327	PTPRN2	0.1483	1.08E-02
cg00616687	SIM2	0.1434	3.38E-02
cg00011482	PAX9	0.1367	1.53E-03
cg23971987		0.1362	3.91E-03
cg12659494	SIM2	0.1335	1.94E-03
cg17799033	PTPRN2	0.1317	1.10E-02
cg07521193		0.1301	2.29E-03
cg18004701	PTPRN2	0.1269	1.01E-02
cg10911990	PAX9	0.1269	1.52E-04
cg06367647	FAM5C	0.1225	6.38E-03
cg20910490		0.1204	1.18E-02
cg01373444	PRR5;PRR5-ARHGAP8	0.1197	1.74E-02
cg14990082		0.1188	2.18E-03
cg27170985		0.1172	1.70E-02
cg04678916		0.1170	3.88E-02

cg27312652	SIM2	0.1156	1.87E-05
cg20673819	C6orf176	0.1149	4.88E-02
cg19040474	RPH3AL	0.1146	5.41E-03
cg02877240	IFRD1	0.1139	6.80E-03
cg03839717		0.1130	1.76E-02
cg12889126	KCNC2	0.1121	1.40E-03
cg24854430	ENPP6	0.1102	2.87E-02
cg15226608	PEX5L	0.1098	2.09E-03
cg14021550		0.1095	1.51E-03
cg26073844	GSDMC	0.1087	2.24E-02
cg08096367		0.1063	1.18E-02
cg05369857		0.1057	1.14E-02
cg04897931	RPH3AL	0.1048	1.82E-02
cg08698198		0.1047	1.01E-02
cg13023870		0.1045	4.88E-02
cg06879222		0.1033	1.40E-03
cg18894200	PTPRN2	0.1031	1.32E-02
cg23655970	PAX9	0.1031	3.74E-02
cg00731944	WSCD1	0.1026	8.11E-03
cg06323479		0.1019	3.12E-02
cg07861448	MSC	-0.1024	2.28E-02
cg00303223	SHOX2	-0.1050	3.83E-03
cg19575372		-0.1050	9.84E-03
cg21847036	SHOX2	-0.1056	5.41E-03
cg24028202	ISLR2	-0.1085	8.42E-05
cg08654915		-0.1085	6.69E-03
cg15726154	SHOX2	-0.1087	2.31E-02
cg10526277		-0.1091	1.62E-02
cg06496728		-0.1092	1.83E-02
cg02579136	WNT11	-0.1100	3.44E-02
cg08787251	EVX1	-0.1124	1.43E-03
cg10445315		-0.1156	7.30E-03
cg21552242	SHOX2	-0.1341	2.29E-03
cg19818890		-0.1383	4.39E-03

cg21437028	SHOX2	-0.1396	1.51E-03
cg25506747	SHOX2	-0.1518	5.66E-03
cg03458172	SHOX2	-0.1540	4.44E-03
cg27397850	EVX1	-0.1639	9.25E-04
cg05825073	EVX1	-0.1689	9.25E-04
cg07104209	EVX1	-0.1757	1.89E-05
cg05401764	SHOX2	-0.2471	4.44E-03
cg05209996		-0.2958	1.66E-03
iMT/oLT			
cg10703826	TBX15	0.3273	3.37E-03
cg25340966	TBX15	0.2922	4.35E-03
cg16990168	TBX15	0.2778	2.90E-03
cg10308785	LOC404266;HOXB6	0.2508	7.92E-03
cg24720355	TBX15	0.2461	2.30E-03
cg24884142	TBX15	0.2277	1.83E-03
cg24672833		0.2241	2.22E-02
cg13655674	TBX15	0.2154	4.78E-03
cg11391335	TBX15	0.2139	5.84E-03
cg18944010	TBX15	0.2120	5.27E-03
cg22820316	TBX15	0.2020	3.39E-03
cg26127662	HOXA3	0.2011	2.30E-03
cg26272623	TBX15	0.1956	2.07E-03
cg10967023		0.1936	1.33E-02
cg03942051	TBX15	0.1935	3.37E-03
cg19730691	TBX15	0.1874	1.83E-03
cg23371746	TBX15	0.1830	4.44E-03
cg08942939	TBX15	0.1829	6.61E-03
cg05172122	TBX15	0.1826	5.14E-03
cg19175386		0.1810	1.03E-02
cg02362103	TBX15	0.1776	4.84E-03
cg17616537	HOXB3	0.1774	5.84E-03
cg24842086	TBX15	0.1767	2.66E-03
cg00466334	TBX15	0.1722	6.48E-03

cg06158650	TBX15	0.1698	5.91E-03
cg25255850		0.1687	7.33E-03
cg05387167	HOXB3	0.1685	1.01E-02
cg22378919	TBX15	0.1683	1.17E-02
cg12664119	TBX15	0.1680	1.96E-03
cg03993743	SIM2	0.1658	1.17E-02
cg06354392		0.1655	1.48E-02
cg07850987	HOXB3	0.1653	8.01E-03
cg09284655		0.1646	9.25E-03
cg07892597	TBX15	0.1641	2.07E-03
cg09789768	TBX15	0.1633	8.39E-03
cg02458062	HOXB3	0.1631	1.42E-02
cg26073844	GSDMC	0.1627	1.34E-03
cg10426234		0.1622	2.24E-03
cg27325152	SIM2	0.1610	1.16E-02
cg18197377		0.1610	2.07E-02
cg23143210		0.1589	5.96E-03
cg07280807		0.1582	1.43E-02
cg04514255	MIR10A	0.1578	9.01E-03
cg22005990		0.1576	3.12E-02
cg08101036	HOXA3	0.1568	2.68E-03
cg19759481	HOXA5	0.1567	1.08E-02
cg07917150	HOXA3	0.1552	2.64E-03
cg01956420	COL4A1;COL4A2	0.1542	1.59E-02
cg16787431	HOXB3	0.1533	8.16E-03
cg18351329		0.1519	2.17E-02
cg16856049		0.1506	2.52E-02
cg18220920		0.1505	1.19E-02
cg04863892	HOXA5	0.1498	1.58E-02
cg14565725	TBX15	0.1496	6.32E-03
cg24854430	ENPP6	0.1493	1.11E-03
cg16551520		0.1486	3.74E-03
cg10831607		0.1481	1.26E-02
cg18533201	GDF6	0.1457	1.87E-03

cg18470839		0.1455	4.53E-02
cg15539318		0.1447	8.32E-03
cg02508651		0.1444	6.77E-03
cg17029354		0.1443	1.66E-02
cg18987410	HEPACAM	0.1422	3.42E-02
cg07061298	HOXA3	0.1414	1.83E-03
cg06395298	HOXB3	0.1414	1.07E-02
cg18761549		0.1404	9.64E-03
cg04800503	HOXB3	0.1396	9.30E-03
cg26872137	UNC5D	0.1384	9.10E-03
cg08297082	SIM2	0.1382	1.23E-02
cg14238959		0.1381	8.99E-03
cg09549073	HOXA5	0.1380	7.11E-03
cg03386144		0.1379	7.85E-03
cg01787574	LMO3	0.1378	8.39E-03
cg12685846	TJP1	0.1376	6.32E-03
cg24144440	TBX15	0.1373	4.44E-03
cg02248486	HOXA5	0.1371	1.89E-02
cg24461304		0.1369	1.27E-02
cg09526758	LMO3	0.1365	7.14E-03
cg02873421	HOXB3	0.1364	1.08E-02
cg10794257	HOXA3	0.1362	2.74E-03
cg09952002	HOXB3	0.1352	1.00E-02
cg10143811	LMO3	0.1351	5.89E-03
cg21608519		0.1348	1.44E-02
cg14285150		0.1345	1.73E-02
cg26916621	MIR10A	0.1344	6.32E-03
cg17161250	C2orf40	0.1343	1.62E-02
cg14884929	MIR10A	0.1342	7.39E-03
cg03760839	TBX15	0.1338	2.37E-03
cg25616547		0.1324	1.83E-02
cg02170386		0.1321	1.37E-02
cg16945186		0.1314	2.88E-02
cg08286181		0.1314	8.01E-03

cg08175609		0.1309	1.00E-02
cg15634747		0.1304	1.55E-02
cg21853871	HOXB3	0.1298	9.82E-03
cg15649236	MIR10A	0.1294	1.08E-02
cg02497558	HOXB1	0.1290	6.62E-03
cg13752649	HOXB1	0.1285	6.98E-03
cg25984344	DOCK1;FAM196A	0.1279	2.91E-02
cg15858239	NKAIN3	0.1279	3.42E-02
cg19728226		0.1273	2.19E-02
cg17484671		0.1261	2.42E-02
cg12128839	HOXA5	0.1258	4.66E-03
cg16937268		0.1251	1.10E-02
cg08149193	ALX4	0.1242	1.86E-02
cg26457248	MBP	0.1242	3.39E-02
cg04903623		0.1241	7.93E-03
cg19094333		0.1241	6.61E-04
cg12910797	HOXB3	0.1240	7.35E-03
cg25877512		0.1235	2.07E-03
cg03807298		0.1228	8.68E-03
cg22799141		0.1224	3.04E-02
cg10530883	IRX1	0.1222	4.19E-02
cg25866143	HOXA5	0.1215	6.62E-03
cg01244015	FAM124B	0.1215	1.42E-02
cg01593673	HOXB3	0.1212	5.91E-03
cg00661970		0.1210	2.04E-02
cg25332502	MBP	0.1207	4.35E-03
cg19104015	HOXB3	0.1203	1.90E-02
cg11971423	HOXB4	0.1198	2.49E-02
cg27475132	FAT1	0.1197	3.97E-02
cg02394746		0.1197	2.00E-02
cg23971987		0.1191	1.37E-02
cg07942135	HOXA3	0.1186	4.01E-03
cg02081305	SULT6B1	0.1185	6.62E-03
cg23348270		0.1184	3.56E-02

cg06245037	ALX4	0.1183	5.89E-03
cg16118212	EFEMP1	0.1179	2.51E-02
cg21207665	PAX9	0.1175	9.31E-03
cg24704177	HOXD3	0.1171	3.87E-02
cg08164294	HOXA3	0.1165	6.92E-03
cg26622699	HOXB3	0.1165	1.18E-02
cg18095938		0.1164	6.61E-04
cg02060566		0.1164	4.08E-02
cg18825414	C18orf2	0.1159	9.18E-03
cg01370449	HOXA5	0.1158	1.19E-02
cg23014425	HOXB3	0.1157	1.10E-02
cg04478428	SLC17A9	0.1157	2.24E-02
cg01833675	FAM124B	0.1157	1.54E-02
cg26611765		0.1155	1.42E-02
cg06982190		0.1153	1.76E-02
cg21156438		0.1149	2.44E-03
cg18940674		0.1149	4.06E-03
cg02470521	HOXD11	0.1147	1.38E-02
cg00921266	HOXA3	0.1147	1.81E-03
cg07077013		0.1146	2.10E-02
cg18776460	LRIG3	0.1144	3.80E-03
cg10165801	HOXB3	0.1142	1.64E-02
cg02086195	DHRS3	0.1140	3.26E-02
cg02749463	HOXB3	0.1139	2.53E-02
cg00040268	HOXA3	0.1139	4.69E-03
cg21045464		0.1136	9.79E-03
cg06186155	HOXB3	0.1136	1.23E-02
cg12544951	PAX1	0.1134	4.16E-03
cg01181415	LMO3	0.1124	3.89E-03
cg19986012	HOXB3	0.1123	6.66E-03
cg10996058	C1QL2	0.1118	1.48E-03
cg10399005		0.1118	4.44E-03
cg14568830		0.1118	1.33E-02
cg05259508		0.1117	2.58E-02

cg13777513		0.1117	3.80E-03
cg27125044		0.1114	2.96E-02
cg00586531		0.1113	4.00E-03
cg19117047		0.1110	9.01E-03
cg06439547	COG2	0.1107	7.52E-03
cg19858749		0.1105	3.63E-02
cg03463818	TMEM67	0.1104	7.93E-03
cg15226608	PEX5L	0.1103	5.36E-03
cg21229570		0.1103	4.66E-02
cg26687072	HOXB1	0.1102	1.15E-02
cg12713060		0.1099	1.03E-02
cg05581701	MT1M	0.1098	4.60E-02
cg24948406	HOXB1	0.1093	7.00E-03
cg02811362		0.1090	1.63E-02
cg07133930		0.1090	2.05E-02
cg20475486		0.1086	1.84E-02
cg15065049	HOXB3	0.1086	9.93E-03
cg27349540		0.1086	3.14E-03
cg13172549	HOXA3	0.1081	2.98E-03
cg20124587	HOXD11	0.1081	5.87E-03
cg18136932	C5orf49	0.1080	1.49E-02
cg23683588	PAX9	0.1079	2.39E-02
cg21978924	ALX4	0.1077	3.01E-02
cg26072749	MIR10A	0.1075	4.03E-02
cg13572309	LMO3	0.1074	5.31E-03
cg26411222		0.1072	2.12E-02
cg08076955	HOXB1	0.1069	6.43E-03
cg05398903		0.1068	1.17E-02
cg20184247	LOC404266;HOXB5	0.1067	7.64E-03
cg00620733	MEOX1	0.1066	1.95E-02
cg20784391		0.1065	4.82E-03
cg02527112	HOXD11	0.1060	1.90E-03
cg22231101	JAKMIP1	0.1058	7.96E-03

cg07387607	BAT2	0.1057	3.66E-02
cg05527785	HOXB4	0.1056	6.62E-03
cg09601175		0.1055	1.78E-02
cg14632140	LMO3	0.1055	5.84E-03
cg24154839	GABRA4	0.1054	2.97E-02
cg20340866		0.1052	2.17E-02
cg22962123	HOXA3	0.1051	3.49E-03
cg02143743	AGBL1	0.1047	1.10E-02
cg25313930	FAM124B	0.1043	2.58E-02
cg20688847		0.1043	3.74E-02
cg24880701	LBXCOR1	0.1042	2.66E-02
cg04132007	KIAA1688	0.1040	4.51E-03
cg12188986	CCDC67	0.1038	2.55E-02
cg00875849		0.1036	4.44E-03
cg19965221	TJP1	0.1036	4.71E-02
cg10584587	PACRG	0.1032	1.78E-02
cg02898094	DLX6AS;DLX6	0.1030	2.54E-02
cg06367647	FAM5C	0.1029	7.48E-03
cg13834623	SCGN	0.1029	1.20E-02
cg14880184	GATA6	0.1027	1.51E-02
cg02574073	LOC404266;HOXB6	0.1026	4.73E-02
cg18482892	ARHGEF3	0.1026	4.57E-02
cg20219381	RGS22	0.1026	2.00E-02
cg24900666	HOXB1	0.1025	1.46E-02
cg20988291		0.1023	2.28E-02
cg07830847	KCNA10	0.1022	2.38E-02
cg15767955		0.1019	2.42E-02
cg02337614		0.1018	1.05E-02
cg13441730	PTPRD	0.1017	3.38E-02
cg18790559		0.1015	3.53E-02
cg17510278	PAX1	0.1014	6.62E-03
cg14006800		0.1011	3.06E-02
cg24516901	FAM124B	0.1011	3.33E-02

cg17569124	HOXA5	0.1010	1.84E-02
cg23130097	MAL2	0.1010	4.54E-02
cg05824218	RARA	0.1003	5.89E-03
cg19975849		0.1002	7.99E-03
cg02184008	ALX4	0.1001	1.09E-02
cg20389635	PTHLH	-0.1000	3.35E-02
cg20273260	LYPD1	-0.1002	9.49E-03
cg12232308	IER3;FLOT1	-0.1004	2.81E-02
cg04258520	MARK3	-0.1005	2.16E-02
cg10575547	TRERF1	-0.1006	4.42E-02
cg13967702	ESRRG	-0.1006	1.47E-02
cg00172872		-0.1007	1.78E-02
cg15681239	DLEC1	-0.1007	1.00E-02
cg14642696	LMX1B	-0.1012	4.56E-02
cg17893669	KLHL26	-0.1014	2.57E-02
cg18767321	JOSD1	-0.1014	6.82E-03
cg00437311		-0.1021	3.98E-02
cg20038591	EMX2	-0.1024	8.16E-03
cg18025409	TNFRSF9	-0.1027	2.23E-02
cg19291696		-0.1033	8.16E-03
cg08327038	CIRH1A	-0.1034	1.33E-02
cg24178897		-0.1037	1.71E-02
cg11958666		-0.1039	2.07E-03
cg00955911	FOXA1	-0.1039	2.58E-02
cg25316429	EMX2OS	-0.1039	1.22E-02
cg23098803		-0.1042	4.02E-03
cg08268692	GDPD4	-0.1043	4.85E-02
cg09969043	NR2F2	-0.1045	1.15E-02
cg03453870		-0.1046	2.98E-02
cg05545914		-0.1046	3.99E-02
cg10435609	MGC27382	-0.1047	3.71E-02
cg20178172	IER3;FLOT1	-0.1049	3.30E-02
cg03887721		-0.1050	2.31E-02
cg13337949	IER3;FLOT1	-0.1051	1.17E-02

cg00865429	CLNK	-0.1058	3.83E-02
cg04389897	TFAP2A	-0.1066	1.26E-02
cg11916478	IQSEC3	-0.1066	4.58E-02
cg25176746	MYF6	-0.1068	2.04E-02
cg21468929	EMX2OS	-0.1069	1.08E-02
cg16717008		-0.1074	4.87E-02
cg19033654	IER3;FLOT1	-0.1079	1.01E-02
cg10371155	MED1	-0.1080	3.40E-02
cg00011856	IGFBP5	-0.1083	1.69E-02
cg26288991	EMX2OS	-0.1083	9.18E-03
cg26570179	OSR2	-0.1089	4.44E-02
cg13158481	HOXD9	-0.1089	2.93E-02
cg12777520	LMX1B	-0.1091	2.93E-02
cg18203366	NR5A2	-0.1092	2.68E-03
cg14223293		-0.1093	3.72E-02
cg08217163		-0.1100	1.23E-02
cg08457178	IER3;FLOT1	-0.1100	1.83E-02
cg17222143	USH2A	-0.1103	5.33E-03
cg04522596		-0.1103	5.96E-03
cg14601868	HOXD8	-0.1105	2.24E-03
cg05925497	FLJ32810	-0.1106	1.42E-02
cg09611490		-0.1108	1.22E-02
cg08193273	TRHR	-0.1110	2.21E-02
cg09235583	IER3;FLOT1	-0.1112	1.10E-02
cg09360044		-0.1112	4.38E-02
cg25004840		-0.1113	2.20E-02
cg16825290	HPSE2	-0.1123	2.35E-02
cg01410923	MYO1B	-0.1123	3.10E-02
cg26956371	C10orf88	-0.1125	2.27E-02
cg25913882	CUBN	-0.1126	3.75E-02
cg14014890	PQLC3	-0.1128	1.86E-02
cg07774193		-0.1134	1.37E-02
cg21661837	TFAP2A	-0.1136	2.91E-02
cg25698741		-0.1139	3.76E-02

cg22624255	ZNF101	-0.1144	1.90E-02
cg02736602	DENND1B	-0.1145	5.84E-03
cg12969193	HOXD9	-0.1146	3.81E-02
cg13461390	ESRRG	-0.1147	1.88E-02
cg07104209	EVX1	-0.1148	1.32E-02
cg20160885	RNF216L	-0.1149	1.88E-02
cg22110158	ST14	-0.1151	2.94E-02
cg10595547		-0.1153	7.79E-03
cg15991405	HOXD9	-0.1161	2.78E-02
cg21097733	HOXB2	-0.1169	1.18E-02
cg04057599		-0.1170	2.61E-02
cg09075968	PCID2	-0.1176	1.83E-02
cg14063191	ZDHHC12	-0.1178	4.44E-03
cg11601932		-0.1178	3.89E-02
cg00756032	MYF6	-0.1180	1.05E-02
cg09579310		-0.1186	3.12E-02
cg12204897	ZDHHC12	-0.1196	1.12E-02
cg09862711		-0.1200	4.07E-03
cg18395466		-0.1202	4.84E-02
cg07248223	CCR7	-0.1205	1.26E-02
cg09307788		-0.1206	5.10E-03
cg26342147	EMX2OS	-0.1213	5.31E-03
cg09287864		-0.1219	1.03E-02
cg19048251		-0.1224	1.30E-02
cg27520776	ESRRG	-0.1229	1.42E-02
cg04102510	ITPKB	-0.1235	3.07E-02
cg03495084	SH3BP5	-0.1236	2.59E-02
cg15689835	LMX1B	-0.1242	1.18E-02
cg23679344	MED1	-0.1243	1.32E-02
cg24573321	BAI3	-0.1253	1.00E-02
cg04911280	AARS2	-0.1254	2.96E-02
cg20348858	EMX2;EMX2OS	-0.1261	2.42E-03
cg05036106	EMX2OS	-0.1266	2.71E-02
cg02053340	PARD6B	-0.1267	3.37E-03

cg24874977		-0.1269	4.42E-02
cg22223119	FARP1	-0.1269	1.08E-02
cg12117227	EXOC2	-0.1269	5.64E-03
cg17573720	HOTAIR	-0.1270	2.28E-02
cg04470054	RPTOR	-0.1271	3.49E-02
cg03321133	HOXD8	-0.1277	4.44E-03
cg14433074	IER3;FLOT1	-0.1277	1.48E-03
cg04421971	EMX2OS	-0.1278	9.29E-03
cg07078958	SH3BP5	-0.1281	2.77E-02
cg17320707	EMX2	-0.1290	8.46E-03
cg20597409	SLC25A21;LOC100 129794	-0.1291	1.54E-02
cg06456154		-0.1296	8.97E-03
cg03221073	HMCN1;MIR548F1	-0.1299	4.05E-02
cg00756451	TBX5	-0.1306	2.74E-02
cg25233271		-0.1308	3.40E-02
cg20406460		-0.1309	5.49E-03
cg13495235	C9orf24	-0.1310	2.77E-02
cg05602356	PDE4D	-0.1311	1.99E-02
cg07833262	EMX2OS	-0.1324	1.02E-02
cg02230593	EMX2OS	-0.1325	1.44E-02
cg21608600		-0.1327	2.81E-02
cg18972885	EMX2OS	-0.1341	1.53E-02
cg04256864	EMX2OS	-0.1361	1.73E-02
cg11124080	EMX2OS	-0.1369	1.73E-02
cg21723903	EFCAB4B	-0.1371	1.44E-02
cg19925204	EMX2	-0.1375	2.53E-03
cg25423004	HOXD8	-0.1401	8.97E-03
cg16747928		-0.1412	1.42E-02
cg12663656	CCDC64	-0.1421	4.47E-03
cg02019574		-0.1423	4.44E-02
cg00718470		-0.1430	5.24E-03
cg20792376	EMX2OS	-0.1431	1.28E-02
cg12623982	EMX2OS	-0.1441	9.18E-03

cg12121660	HOXB2	-0.1442	2.04E-02
cg13979277	LMX1B	-0.1458	6.61E-03
cg18516557		-0.1458	3.08E-02
cg11248896		-0.1470	8.46E-03
cg16234557	EMX2OS	-0.1477	7.35E-03
cg13445608		-0.1486	3.40E-02
cg10846980	EMX2;EMX2OS	-0.1486	3.37E-03
cg08124027	KHSRP	-0.1486	2.84E-03
cg09334277		-0.1492	4.16E-03
cg25793931		-0.1502	5.10E-03
cg06896987		-0.1506	9.93E-03
cg18700744	NAA25	-0.1514	3.66E-02
cg18784943		-0.1529	2.18E-02
cg03701930		-0.1536	6.61E-04
cg11195065	EMX2OS	-0.1536	1.11E-02
cg26389913		-0.1553	7.35E-03
cg24725789		-0.1561	4.35E-03
cg23229261	OTX1	-0.1563	3.63E-02
cg21472506	OTX1	-0.1586	2.47E-02
cg03311684	EMX2OS	-0.1586	8.79E-03
cg08451832		-0.1609	1.94E-02
cg08406102	EMX2OS	-0.1623	4.19E-03
cg25556690		-0.1650	3.40E-02
cg27630311	TBX3	-0.1652	2.39E-02
cg18561589	EMX2OS	-0.1658	1.05E-02
cg22920873	C7orf55	-0.1663	1.91E-03
cg11375458		-0.1668	1.08E-02
cg16489193	VPS52;RPS18	-0.1679	9.79E-03
cg17147211		-0.1686	4.67E-02
cg06141846	EMX2OS	-0.1723	6.65E-03
cg04368796	NKIRAS2	-0.1804	1.19E-02
cg13630043	EMX2	-0.1878	4.10E-03
cg08384314	IER3;FLOT1	-0.1996	4.16E-03
cg15526081		-0.2232	6.21E-03

cg04693928	LMX1B	-0.2299	9.58E-03
cg19346371	TBX3	-0.2699	1.96E-03
iMT/iLT			
cg19346371	TBX3	-0.2622	4.91E-03
cg23229261	OTX1	-0.2367	5.50E-03
cg24039697	FAM198B	-0.2256	1.14E-02
cg21472506	OTX1	-0.2082	8.00E-03
cg15526081		-0.2056	1.65E-02
cg04937416	PTPRN2	-0.1807	8.74E-03
cg13630043	EMX2	-0.1758	9.97E-03
cg09334277		-0.1744	4.18E-03
cg03301200		-0.1742	5.92E-03
cg02019574		-0.1726	3.96E-02
cg08451832		-0.1726	3.34E-02
cg03993743	SIM2	-0.1681	3.35E-02
cg18561589	EMX2OS	-0.1675	1.44E-02
cg06141846	EMX2OS	-0.1660	1.72E-02
cg03311684	EMX2OS	-0.1615	8.61E-03
cg27630311	TBX3	-0.1611	3.08E-02
cg21608600		-0.1610	1.81E-02
cg00756451	TBX5	-0.1609	1.60E-03
cg12121660	HOXB2	-0.1579	9.97E-03
cg15572489	PTPRN2	-0.1562	1.55E-02
cg24725789		-0.1561	1.20E-02
cg11195065	EMX2OS	-0.1548	1.40E-02
cg03304437	FAM198B	-0.1526	4.80E-03
cg24811290	FAM198B	-0.1519	4.00E-03
cg07521193		-0.1510	1.18E-03
cg23558626		-0.1509	2.95E-03
cg19048251		-0.1501	6.72E-03
cg25793931		-0.1500	1.07E-02
cg07184578		-0.1484	1.58E-02
cg11124080	EMX2OS	-0.1471	1.53E-02
cg11536474		-0.1399	3.75E-02

cg08406102	EMX2OS	-0.1397	1.66E-02
cg09307788		-0.1386	3.99E-03
cg16713262		-0.1386	1.40E-02
cg20792376	EMX2OS	-0.1383	2.62E-02
cg10846980	EMX2;EMX2OS	-0.1374	1.11E-02
cg19925204	EMX2	-0.1373	6.29E-03
cg16234557	EMX2OS	-0.1340	2.15E-02
cg08297082	SIM2	-0.1333	1.23E-02
cg02162886	SIX1	-0.1333	2.81E-02
cg27325152	SIM2	-0.1325	2.67E-02
cg18972885	EMX2OS	-0.1320	2.19E-02
cg09568355		-0.1317	3.45E-02
cg07833262	EMX2OS	-0.1307	2.86E-02
cg02230593	EMX2OS	-0.1298	3.64E-02
cg07577267	OCA2	-0.1297	2.34E-02
cg20348858	EMX2;EMX2OS	-0.1293	1.63E-03
cg20406460		-0.1279	2.20E-02
cg06456154		-0.1277	1.53E-02
cg12623982	EMX2OS	-0.1264	2.81E-02
cg24573321	BAI3	-0.1263	1.69E-03
cg00756032	MYF6	-0.1237	2.06E-02
cg24796272		-0.1237	7.14E-03
cg26295921	PTPRN2	-0.1231	3.09E-02
cg10720723	PTPRN2	-0.1227	4.19E-02
cg04317962		-0.1221	4.38E-03
cg04522596		-0.1213	2.41E-03
cg10595547		-0.1196	1.07E-02
cg03883295	EN1	-0.1196	1.59E-02
cg00718470		-0.1191	3.97E-02
cg25176746	MYF6	-0.1184	3.35E-02
cg17320707	EMX2	-0.1183	3.45E-02
cg21097733	HOXB2	-0.1164	2.19E-02
cg14757661		-0.1161	1.53E-02
cg27520776	ESRRG	-0.1158	3.89E-02

cg09160681		-0.1154	4.29E-04
cg02767633		-0.1152	1.53E-02
cg16170767		-0.1147	2.15E-02
cg26342147	EMX2OS	-0.1140	9.97E-03
cg10122865	OTX1	-0.1139	1.46E-02
cg18391978		-0.1106	2.35E-02
cg13979277	LMX1B	-0.1104	3.35E-02
cg08096367		-0.1097	4.92E-03
cg07920365		-0.1091	7.15E-03
cg17222143	USH2A	-0.1080	2.67E-02
cg26288991	EMX2OS	-0.1075	3.35E-02
cg04421971	EMX2OS	-0.1070	3.03E-02
cg19241327	PTPRN2	-0.1068	3.40E-02
cg08193273	TRHR	-0.1060	4.72E-02
cg22030072	EN1	-0.1051	2.07E-02
cg19047660		-0.1048	5.00E-02
cg05141014	PART1	-0.1047	5.04E-03
cg03495498		-0.1042	6.87E-03
cg04524478	MYF5	-0.1041	6.87E-03
cg05000331		-0.1038	5.52E-03
cg10838157		-0.1036	1.87E-03
cg02877240	IFRD1	-0.1036	1.16E-02
cg03140412	SIGIRR	-0.1036	4.24E-02
cg26023087		-0.1032	1.22E-02
cg13967702	ESRRG	-0.1015	3.23E-02
cg19351026		-0.1011	4.91E-03
cg01373444	PRR5	-0.1011	4.76E-02
cg27209571	DNER	-0.1006	1.69E-02
cg01524860		-0.1004	2.27E-02
cg18203366	NR5A2	-0.1000	9.97E-03
cg08076955	HOXB1	0.1001	8.50E-03
cg15908709	LOC404266	0.1005	2.41E-03
cg13172549	HOXA3	0.1006	7.02E-03
cg16605327	TBX5	0.1017	2.30E-02

cg02362103	TBX15	0.1019	2.05E-02
cg24948406	HOXB1	0.1027	1.78E-02
cg20184247	LOC404266	0.1030	1.51E-02
cg22187251	GCNT2	0.1031	3.96E-02
cg07133930		0.1032	3.85E-02
cg12002589	LHX2	0.1038	4.04E-02
cg12910797	HOXB3	0.1041	3.31E-02
cg11502555		0.1041	1.71E-02
cg11809614	C1orf156	0.1042	5.50E-03
cg12570134	HOXB3	0.1043	3.42E-02
cg26687072	HOXB1	0.1045	3.03E-02
cg22962123	HOXA3	0.1045	9.94E-03
cg19728226		0.1052	3.58E-02
cg19164987	HOXC4;HOXC5;HOXC6	0.1056	3.82E-03
cg14230696		0.1059	4.53E-02
cg19536929		0.1062	1.69E-02
cg19975849		0.1066	1.72E-02
cg26566121		0.1067	1.95E-04
cg18949192		0.1076	2.17E-02
cg19794507		0.1076	1.69E-02
cg24900666	HOXB1	0.1079	1.83E-02
cg07981495	CGA	0.1089	1.63E-03
cg10877086		0.1098	1.32E-02
cg12713060		0.1102	2.85E-02
cg15325373	EMX2	0.1105	2.32E-02
cg12153794		0.1105	6.79E-03
cg08942939	TBX15	0.1110	4.43E-02
cg16551520		0.1113	1.46E-02
cg05172122	TBX15	0.1119	4.93E-02
cg02311193		0.1120	7.14E-03
cg22053945	HOXB3	0.1123	2.82E-03
cg12664119	TBX15	0.1125	3.03E-03
cg00921266	HOXA3	0.1128	3.36E-03

cg23442672		0.1130	1.02E-02
cg15964309		0.1135	3.77E-02
cg20489228		0.1140	9.97E-03
cg00507008		0.1141	1.09E-02
cg25032089	HOXB3	0.1146	2.27E-02
cg18533201	GDF6	0.1146	9.97E-03
cg09601584	LOC404266	0.1148	3.25E-03
cg01396176		0.1149	8.77E-03
cg16609260		0.1151	8.32E-03
cg26611765		0.1162	1.15E-02
cg05398903		0.1163	3.91E-02
cg02749463	HOXB3	0.1164	3.34E-02
cg08106887	HOXC4;HOXC5;HOXC6	0.1172	3.08E-03
cg25768734	HOXA3	0.1174	3.82E-03
cg21045464		0.1175	1.12E-02
cg03942051	TBX15	0.1176	1.32E-02
cg00998124		0.1178	3.68E-02
cg19986012	HOXB3	0.1182	7.15E-03
cg20340866		0.1197	1.62E-02
cg10426234		0.1200	2.92E-03
cg03257417		0.1203	2.16E-02
cg22773899		0.1203	1.63E-03
cg06912966		0.1209	7.86E-03
cg08286181		0.1209	3.60E-02
cg06982190		0.1210	1.40E-02
cg07006091		0.1212	1.87E-03
cg09417809		0.1217	9.97E-03
cg23014425	HOXB3	0.1219	3.85E-03
cg18944010	TBX15	0.1223	3.40E-02
cg12437821		0.1236	4.91E-03
cg26272623	TBX15	0.1241	4.38E-03
cg09941406		0.1246	1.07E-02
cg08164294	HOXA3	0.1248	2.15E-02

cg14639847		0.1248	1.65E-02
cg15065049	HOXB3	0.1249	4.38E-03
cg06186155	HOXB3	0.1250	6.28E-03
cg26622699	HOXB3	0.1252	7.82E-03
cg14123543		0.1258	2.36E-03
cg19104015	HOXB3	0.1265	2.86E-02
cg21608519		0.1268	7.02E-03
cg22820316	TBX15	0.1273	7.06E-03
cg26343001		0.1279	6.56E-03
cg13752649	HOXB1	0.1281	9.50E-03
cg24720355	TBX15	0.1282	1.81E-02
cg11391335	TBX15	0.1287	1.82E-02
cg02508651		0.1295	3.41E-02
cg01593673	HOXB3	0.1299	4.38E-03
cg21853871	HOXB3	0.1305	9.97E-03
cg05574357		0.1312	7.82E-03
cg02497558	HOXB1	0.1315	5.49E-03
cg07892597	TBX15	0.1321	2.41E-03
cg11971423	HOXB4	0.1329	2.15E-02
cg21481275		0.1340	8.58E-03
cg09952002	HOXB3	0.1345	1.59E-02
cg07942135	HOXA3	0.1355	4.38E-03
cg12205669		0.1360	5.75E-04
cg04903623		0.1367	8.50E-03
cg23143210		0.1375	1.53E-02
cg15649236	MIR10A	0.1402	3.25E-03
cg14285150		0.1421	9.97E-03
cg10165801	HOXB3	0.1424	2.88E-03
cg18095938		0.1427	1.95E-04
cg02873421	HOXB3	0.1432	7.14E-03
cg08101036	HOXA3	0.1433	1.41E-02
cg20688847		0.1435	1.86E-02
cg17029354		0.1446	3.64E-02
cg07061298	HOXA3	0.1447	1.63E-03

cg10794257	HOXA3	0.1451	1.53E-02
cg05128922		0.1453	2.51E-03
cg00040268	HOXA3	0.1456	3.03E-03
cg16990168	TBX15	0.1456	1.14E-02
cg14187844		0.1463	2.27E-03
cg04800503	HOXB3	0.1465	2.41E-03
cg24842086	TBX15	0.1465	1.35E-03
cg00603371	EMX2;EMX2OS	0.1493	3.90E-02
cg06354392		0.1511	2.81E-02
cg16787431	HOXB3	0.1524	7.14E-03
cg14884929	MIR10A	0.1526	1.63E-03
cg02824386		0.1533	1.63E-03
cg07917150	HOXA3	0.1539	1.55E-02
cg26411222		0.1543	2.51E-03
cg15539318		0.1550	2.01E-02
cg24884142	TBX15	0.1555	1.60E-03
cg14411103		0.1560	4.00E-03
cg26916621	MIR10A	0.1560	1.89E-03
cg19858749		0.1587	1.18E-03
cg07533951		0.1589	2.27E-03
cg03386144		0.1596	3.60E-03
cg07850987	HOXB3	0.1598	1.34E-02
cg06395298	HOXB3	0.1608	2.27E-03
cg10831607		0.1618	6.37E-03
cg22005990		0.1625	1.46E-02
cg18761549		0.1638	9.97E-03
cg02060566		0.1653	1.35E-03
cg16945186		0.1674	2.46E-02
cg07541701		0.1680	2.41E-03
cg25616547		0.1682	4.92E-03
cg25255850		0.1690	3.82E-03
cg08175609		0.1698	8.71E-03
cg03532904		0.1699	2.41E-03
cg06721255		0.1710	3.25E-03

cg05387167	HOXB3	0.1730	4.92E-03
cg04514255	MIR10A	0.1731	7.20E-03
cg24461304		0.1734	4.91E-03
cg22799141		0.1757	1.63E-03
cg20307896		0.1781	1.97E-03
cg16856049		0.1804	9.49E-03
cg05259508		0.1810	3.99E-03
cg17616537	HOXB3	0.1825	3.66E-03
cg07080050	HOXC4;HOXC5;HOXC6	0.1838	3.96E-04
cg02458062	HOXB3	0.1878	4.02E-03
cg21229570		0.1878	9.06E-03
cg15772924	HOXC4;HOXC5;HOXC6	0.1900	1.95E-04
cg18470839		0.1913	5.75E-04
cg18220920		0.1945	3.60E-03
cg01529365		0.2014	2.11E-03
cg18197377		0.2036	5.66E-03
cg14283944		0.2045	2.52E-03
cg19175386		0.2107	4.38E-03
cg24208826		0.2131	1.16E-02
cg25340966	TBX15	0.2174	3.92E-03
cg10308785	LOC404266	0.2264	1.89E-02
cg26127662	HOXA3	0.2267	2.79E-03
cg24672833		0.2452	1.92E-02
cg10703826	TBX15	0.2593	2.98E-03

Supplementary table 2: Gene ontology of DMGs in iMT/oLT, iMT/iLT of subchondral bone and shared DMGs in iMT/oLT of subchondral bone and cartilage.

	iMT/oLT_sub		iMT/iLT_sub		iMT/oLT_sub&cartilage	
GO Terms	Fold (1)	P (2)	Fold	P	Fold	p
GO:0009952~anterior/posterior pattern	21.1	3.79E-16	/	/	32.21	1.04E-10

formation						
GO:0003002~regionalization	16.58	2.02E-16	/	/		
GO:0003002~regionalization	/	/	/	/	24.97	1.79E-10
GO:0007389~pattern specification process	13.39	1.76E-16	28.31	4.25E-20	18.42	8.23E-10
GO:0048705~skeletal system morphogenesis	23.6	1.80E-15	28.42	2.45E-07	36.60	3.02E-10
GO:0048704~embryonic skeletal system morphogenesis	35.46	1.42E-13	55.84	2.45E-09	64.73	7.40E-11
GO:0048706~embryonic skeletal system development	26.25	4.97E-12	51.67	1.56E-11	47.91	5.48E-10
GO:0048562~embryonic organ morphogenesis	17.54	7.52E-12	29.92	9.61E-10	27.74	3.33E-08
GO:0048568~embryonic organ development	14.46	1.38E-11	23.13	7.49E-09	21.45	1.64E-07
GO:0048598~embryonic morphogenesis	9.62	3.77E-11	/	/	13.35	7.42E-07
GO:0001501~skeletal system development	/	/	/	/	14.14	9.01E-08
GO:0043009~chordate embryonic development	/	/	/	/	13.62	1.14E-07
GO:0009792~embryonic development ending in birth or egg hatching	/	/	/	/	13.50	1.12E-07
GO:0043565~sequence-specific DNA binding	6.72	1.08E-12	13.11	1.17E-15	8.69	1.98E-07
GO:0003700~transcription factor activity	4.95	2.38E-12	9.45	7.62E-16	6.66	7.64E-08
GO:0006355~regulation of transcription, DNA-dependent	3.16	9.47E-09	4.94	9.96E-10	3.93	1.28E-05
dna-binding	3.23	1.12E-08	5.81	6.58E-11	4.49	6.54E-06
GO:0051252~regulation of RNA metabolic process	3.09	1.64E-08	4.83	1.26E-09	3.84	1.64E-05
GO:0030528~transcription regulator activity	3.29	3.05E-08	6.09	1.93E-12	4.29	9.69E-06
GO:0045449~regulation of transcription	2.39	1.12E-06	3.67	1.28E-08	2.84	3.75E-04
GO:0003677~DNA binding	2.4	6.32E-06	3.95	7.17E-09	2.96	3.51E-04
transcription regulation	2.66	1.06E-05	4.38	1.34E-06	3.65	2.64E-04
nucleus	/	/	2.53	1.02E-04		
DNA-binding region:Homeobox	/	/	/	/	25.79	1.76E-08
GO:0045935~positive regulation of	4.98	4.84E-07	6.38	2.69E-04	5.91	1.34E-03

nucleobase, nucleoside, nucleotide and nucleic acid metabolic process						
GO:0045941~positive regulation of transcription	5.24	5.77E-07	7.05	1.38E-04	6.54	7.20E-04
GO:0051173~positive regulation of nitrogen compound metabolic process	4.83	7.11E-07	6.18	3.31E-04	5.73	1.60E-03
GO:0010628~positive regulation of gene expression	5.09	8.11E-07	6.85	1.66E-04	6.35	8.47E-04
GO:0031328~positive regulation of cellular biosynthetic process	4.54	1.55E-06	5.81	4.98E-04	5.39	2.02E-03
GO:0045893~positive regulation of transcription, DNA-dependent	5.54	1.54E-06	/	/	6.88	1.62E-03
GO:0051254~positive regulation of RNA metabolic process	5.5	1.66E-06	/	/	6.82	1.64E-03
GO:0009891~positive regulation of biosynthetic process	4.47	1.70E-06	5.72	5.17E-04	5.31	2.15E-03
GO:0010557~positive regulation of macromolecule biosynthetic process	4.52	3.53E-06	6.08	3.59E-04	5.64	1.57E-03
GO:0045944~positive regulation of transcription from RNA polymerase II promoter	5.45	9.88E-05	/	/		
GO:0010604~positive regulation of macromolecule metabolic process	3.45	1.38E-04	/	/	4.31	7.80E-03
Transcription	2.44	1.40E-04	3.81	4.79E-05	3.57	2.83E-04
GO:0006350~transcription	2.29	1.75E-04	3.03	7.44E-04	2.93	1.89E-03
GO:0021602~cranial nerve morphogenesis	70.68	1.44E-05	144.68	5.90E-05	186.34	2.68E-07
GO:0021545~cranial nerve development	40.92	1.36E-04	83.76	2.77E-04	107.88	2.67E-06
short sequence motif:Antp-type hexapeptide	/	/	/	/	106.54	1.01E-05
GO:0021675~nerve development	31.1	3.71E-04	63.66	5.25E-04	81.99	8.04E-06
GO:0060173~limb development	12.08	1.02E-04	/	/	/	/

GO:0048736~appendage development	12.08	1.02E-04	/	/	/	/
GO:0030326~embryonic limb morphogenesis	12.51	3.85E-04	/	/	/	/
GO:0035113~embryonic appendage morphogenesis	12.51	3.85E-04	/	/	/	/
GO:0035115~embryonic forelimb morphogenesis	/	/	62.82	1.11E-02	64.73	1.01E-02
GO:0035136~forelimb morphogenesis	/	/	54.26	1.46E-02	55.90	1.32E-02
GO:0035108~limb morphogenesis	10.99	7.82E-04	/	/	/	/
GO:0035107~appendage morphogenesis	10.99	7.82E-04	/	/	/	/
developmental protein	/	/	/	/	7.60	5.28E-06
GO:0030878~thyroid gland development	51.83	0.001	132.63	7.42E-05	102.48	4.21E-03
GO:0021615~glossopharyngeal nerve morphogenesis	155.5	0.0022	/	/	409.94	3.45E-04
GO:0021563~glossopharyngeal nerve development	155.5	0.0022	/	/	409.94	3.45E-04
GO:0030324~lung development	9.42	0.0072	/	/	/	/
GO:0030323~respiratory tube development	9.15	0.0079	/	/	/	/
GO:0060541~respiratory system development	8.64	0.01	/	/	/	/
GO:0014706~striated muscle tissue development	7.84	0.015	16.72	2.78E-03	/	/
GO:0060537~muscle tissue development	7.46	0.017	15.92	3.26E-03	/	/
GO:0060538~skeletal muscle organ development	/	/	24.11	7.03E-03	/	/
GO:0007517~muscle organ development	5.16	0.031	9.43	1.82E-02	/	/
GO:0007519~skeletal muscle tissue development	/	/	24.11	7.03E-03	/	/
GO:0048729~tissue morphogenesis	/	/	11.05	1.09E-02	/	/
GO:0002009~morphogenesis of an epithelium	/	/	15.76	2.01E-02	/	/
GO:0008595~determination of anterior/posterior axis, embryo	66.64	0.013	/	/	/	/

GO:0007351~tripartite regional subdivision	66.64	0.013	/	/	/	/
GO:0007350~blastoderm segmentation	58.31	0.016	/	/	/	/

(1) Fold: fold enrichement. (2) P: Benjiamini-adjusted P value. / : not enriched or not significant.

Supplementary table 3: A cluster of transcription regulators were identified differentially methylated. Red ones belong to homeobox superfamily.

**iLT/oLT**	
EVX1	even-skipped homeobox 1
MSC	musculin
PAX9	paired box 9
SHOX2	short stature homeobox 2
SIM2	single-minded family bHLH transcription factor 2
**iMT/oLT**	
ALX4	ALX homeobox 4
DLX6	distal-less homeobox 6
EMX2	empty spiracles homeobox 2
EVX1	even-skipped homeobox 1
FOXA1	forkhead box A1
GATA6	GATA binding protein 6
HOXA3	homeobox A3
HOXA5	homeobox A5
HOXB1	homeobox B1
HOXB2	homeobox B2
HOXB3	homeobox B3
HOXB4	homeobox B4
HOXB6	homeobox B6
HOXD11	homeobox D11
HOXD3	homeobox D3
HOXD8	homeobox D8
HOXD9	homeobox D9
IRX1	iroquois homeobox 1
LMX1B	LIM homeobox transcription factor 1, beta
MED1	mediator complex subunit 1
MEOX1	mesenchyme homeobox 1

MYF6	myogenic factor 6 (herculin)
OSR2	odd-skipped related transciption factor 2
OTX1	orthodenticle homeobox 1
PAX1	paired box 1
PAX9	paired box 9
PCID2	PCI domain containing 2
SIM2	single-minded family bHLH transcription factor 2
TBX15	T-box 15
TBX3	T-box 3
TBX5	T-box 5
TFAP2A	transcription factor AP-2 alpha (activating enhancer binding protein 2 alpha)
TRERF1	transcriptional regulating factor 1
**iMT/iLT**	
EMX2	empty spiracles homeobox 2
EN1	engrailed homeobox 1
HOXA3	homeobox A3
HOXB1	homeobox B1
HOXB2	homeobox B2
HOXB3	homeobox B3
HOXB4	homeobox B4
HOXC4	homeobox C4
HOXC5	homeobox C5
HOXC6	homeobox C6
LHX2	LIM homeobox 2
LMX1B	LIM homeobox transcription factor 1 beta
MYF5	myogenic factor 5
MYF6	myogenic factor 6
OTX1	orthodenticle homeobox 1
SIM2	single-minded family bHLH transcription factor 2
SIX1	SIX homeobox 1
TBX15	T-box 15
TBX3	T-box 3
TBX5	T-box 5

Supplementary table 4: Genes including multiple DMPs

Illumina probes	Associated gene	Mean Δβ	FDR-p value	Location
iLT/oLT				
cg07104209	*EVX1*	-0.1757	1.887E-05	TSS1500
cg05825073	*EVX1*	-0.1689	9.247E-04	1stExon;5'UTR
cg27397850	*EVX1*	-0.1639	9.247E-04	TSS1500
cg05825073	*EVX1*	-0.1689	3.639E-08	1stExon;5'UTR
cg05401764	*SHOX2*	-0.2471	4.435E-03	3'UTR
cg03458172	*SHOX2*	-0.1540	4.435E-03	3'UTR
cg25506747	*SHOX2*	-0.1518	5.662E-03	3'UTR
cg21437028	*SHOX2*	-0.1396	1.505E-03	Body
cg21552242	*SHOX2*	-0.1341	2.289E-03	3'UTR
cg15726154	*SHOX2*	-0.1087	2.314E-02	Body
cg21847036	*SHOX2*	-0.1056	5.410E-03	Body
cg00303223	*SHOX2*	-0.1050	3.834E-03	3'UTR
cg25977493	*SHOX2*	0.1533	6.067E-03	1stExon;5'UTR
cg27312652	*SIM2*	0.1156	1.868E-05	Body
cg12659494	*SIM2*	0.1335	1.938E-03	Body
cg00616687	*SIM2*	0.1434	3.377E-02	1stExon
cg22976224	*SIM2*	0.1951	1.310E-02	TSS1500
cg08297082	*SIM2*	0.2715	2.517E-06	Body
cg27325152	*SIM2*	0.2935	1.868E-05	Body
cg03993743	*SIM2*	0.3339	2.517E-06	Body
cg23655970	*PAX9*	0.1031	3.739E-02	TSS200
cg10911990	*PAX9*	0.1269	1.517E-04	5'UTR
cg00011482	*PAX9*	0.1367	1.526E-03	5'UTR
cg04415798	*PAX9*	0.1523	4.750E-03	5'UTR
cg23683588	*PAX9*	0.1707	8.148E-03	Body
cg21207665	*PAX9*	0.1741	5.662E-03	5'UTR
cg18894200	*PTPRN2*	0.1031	1.324E-02	Body
cg18004701	*PTPRN2*	0.1269	1.009E-02	Body
cg17799033	*PTPRN2*	0.1317	1.103E-02	Body

cg19241327	*PTPRN2*	0.1483	1.075E-02	Body
cg10720723	*PTPRN2*	0.1503	2.506E-03	Body
cg26295921	*PTPRN2*	0.1504	9.874E-03	Body
cg15572489	*PTPRN2*	0.1841	4.091E-03	Body
cg04937416	*PTPRN2*	0.1884	1.137E-02	Body
iMT/oLT				
cg13630043	*EMX2*	-0.1878	4.099E-03	3'UTR
cg19925204	*EMX2*	-0.1375	2.531E-03	3'UTR;Body
cg17320707	*EMX2*	-0.1290	8.464E-03	3'UTR;3'UTR
cg20038591	*EMX2*	-0.1024	8.164E-03	Body
cg10846980	*EMX2;EMX2OS*	-0.1486	3.370E-03	Body;TSS1500
cg20348858	*EMX2;EMX2OS*	-0.1261	2.421E-03	Body;TSS1500
cg06141846	*EMX2OS*	-0.1723	6.647E-03	Body
cg18561589	*EMX2OS*	-0.1658	1.050E-02	Body
cg08406102	*EMX2OS*	-0.1623	4.185E-03	Body
cg03311684	*EMX2OS*	-0.1586	8.787E-03	Body
cg11195065	*EMX2OS*	-0.1536	1.110E-02	Body
cg16234557	*EMX2OS*	-0.1477	7.354E-03	Body
cg12623982	*EMX2OS*	-0.1441	9.179E-03	Body
cg20792376	*EMX2OS*	-0.1431	1.278E-02	Body
cg11124080	*EMX2OS*	-0.1369	1.726E-02	Body
cg04256864	*EMX2OS*	-0.1361	1.730E-02	Body
cg18972885	*EMX2OS*	-0.1341	1.535E-02	Body
cg02230593	*EMX2OS*	-0.1325	1.436E-02	Body
cg07833262	*EMX2OS*	-0.1324	1.017E-02	Body
cg04421971	*EMX2OS*	-0.1278	9.289E-03	Body
cg05036106	*EMX2OS*	-0.1266	2.709E-02	Body
cg26342147	*EMX2OS*	-0.1213	5.312E-03	Body
cg26288991	*EMX2OS*	-0.1083	9.184E-03	Body
cg21468929	*EMX2OS*	-0.1069	1.083E-02	Body
cg25316429	*EMX2OS*	-0.1039	1.217E-02	Body
cg27520776	*ESRRG*	-0.1229	1.421E-02	TSS1500
cg13461390	*ESRRG*	-0.1147	1.876E-02	TSS1500
cg13967702	*ESRRG*	-0.1006	1.475E-02	TSS1500

cg25423004	*HOXD8*	-0.1401	8.974E-03	TSS1500
cg03321133	*HOXD8*	-0.1277	4.442E-03	TSS1500
cg14601868	*HOXD8*	-0.1105	2.241E-03	TSS1500
cg15991405	*HOXD9*	-0.1161	2.784E-02	Body
cg12969193	*HOXD9*	-0.1146	3.806E-02	Body
cg13158481	*HOXD9*	-0.1089	2.929E-02	Body
cg08384314	*IER3;FLOT1*	-0.1996	4.161E-03	3'UTR;TSS1500
cg14433074	*IER3;FLOT1*	-0.1277	1.476E-03	3'UTR;TSS1500
cg09235583	*IER3;FLOT1*	-0.1112	1.097E-02	3'UTR;TSS1500
cg08457178	*IER3;FLOT1*	-0.1100	1.830E-02	3'UTR;TSS1500
cg19033654	*IER3;FLOT1*	-0.1079	1.008E-02	3'UTR;TSS1500
cg13337949	*IER3;FLOT1*	-0.1051	1.173E-02	3'UTR;TSS1500
cg20178172	*IER3;FLOT1*	-0.1049	3.297E-02	3'UTR;TSS1500
cg12232308	*IER3;FLOT1*	-0.1004	2.811E-02	3'UTR;TSS1500
cg04693928	*LMX1B*	-0.2299	9.579E-03	Body
cg13979277	*LMX1B*	-0.1458	6.611E-03	Body
cg15689835	*LMX1B*	-0.1242	1.183E-02	Body
cg12777520	*LMX1B*	-0.1091	2.929E-02	Body
cg14642696	*LMX1B*	-0.1012	4.558E-02	Body
cg02184008	*ALX4*	0.1001	1.093E-02	TSS1500
cg21978924	*ALX4*	0.1077	3.011E-02	TSS1500
cg06245037	*ALX4*	0.1183	5.887E-03	TSS1500
cg08149193	*ALX4*	0.1242	1.864E-02	TSS1500
cg24516901	*FAM124B*	0.1011	3.329E-02	TSS200
cg25313930	*FAM124B*	0.1043	2.575E-02	TSS200
cg01833675	*FAM124B*	0.1157	1.538E-02	TSS200
cg01244015	*FAM124B*	0.1215	1.421E-02	TSS200
cg22962123	*HOXA3*	0.1051	3.491E-03	5'UTR;1stExon
cg13172549	*HOXA3*	0.1081	2.976E-03	5'UTR;TSS200
cg00040268	*HOXA3*	0.1139	4.687E-03	5'UTR
cg00921266	*HOXA3*	0.1147	1.806E-03	5'UTR;TSS200
cg08164294	*HOXA3*	0.1165	6.923E-03	5'UTR;TSS1500
cg07942135	*HOXA3*	0.1186	4.010E-03	5'UTR;TSS1500
cg10794257	*HOXA3*	0.1362	2.739E-03	5'UTR;TSS1500

cg07061298	*HOXA3*	0.1414	1.828E-03	5'UTR;TSS1500
cg07917150	*HOXA3*	0.1552	2.642E-03	5'UTR;TSS1500
cg08101036	*HOXA3*	0.1568	2.684E-03	5'UTR;TSS200
cg26127662	*HOXA3*	0.2011	2.297E-03	5'UTR;TSS1500
cg17569124	*HOXA5*	0.1010	1.844E-02	TSS1500
cg01370449	*HOXA5*	0.1158	1.190E-02	TSS200
cg25866143	*HOXA5*	0.1215	6.616E-03	5'UTR;1stExon
cg12128839	*HOXA5*	0.1258	4.663E-03	TSS200
cg02248486	*HOXA5*	0.1371	1.894E-02	1stExon
cg09549073	*HOXA5*	0.1380	7.114E-03	5'UTR;1stExon
cg04863892	*HOXA5*	0.1498	1.585E-02	TSS200
cg19759481	*HOXA5*	0.1567	1.082E-02	TSS200
cg24900666	*HOXB1*	0.1025	1.460E-02	TSS200
cg08076955	*HOXB1*	0.1069	6.429E-03	TSS200
cg24948406	*HOXB1*	0.1093	7.001E-03	5'UTR;1stExon
cg26687072	*HOXB1*	0.1102	1.153E-02	TSS200
cg13752649	*HOXB1*	0.1285	6.977E-03	TSS1500
cg02497558	*HOXB1*	0.1290	6.616E-03	TSS1500
cg15065049	*HOXB3*	0.1086	9.927E-03	Body
cg19986012	*HOXB3*	0.1123	6.657E-03	Body
cg06186155	*HOXB3*	0.1136	1.231E-02	5'UTR
cg02749463	*HOXB3*	0.1139	2.530E-02	5'UTR
cg10165801	*HOXB3*	0.1142	1.638E-02	5'UTR
cg23014425	*HOXB3*	0.1157	1.097E-02	5'UTR
cg26622699	*HOXB3*	0.1165	1.181E-02	Body
cg19104015	*HOXB3*	0.1203	1.902E-02	5'UTR
cg01593673	*HOXB3*	0.1212	5.906E-03	5'UTR
cg12910797	*HOXB3*	0.1240	7.354E-03	5'UTR;1stExon
cg21853871	*HOXB3*	0.1298	9.822E-03	Body
cg09952002	*HOXB3*	0.1352	1.003E-02	5'UTR
cg02873421	*HOXB3*	0.1364	1.082E-02	Body
cg04800503	*HOXB3*	0.1396	9.300E-03	5'UTR
cg06395298	*HOXB3*	0.1414	1.069E-02	5'UTR;1stExon
cg16787431	*HOXB3*	0.1533	8.160E-03	Body

cg02458062	*HOXB3*	0.1631	1.424E-02	Body
cg07850987	*HOXB3*	0.1653	8.008E-03	5'UTR
cg05387167	*HOXB3*	0.1685	1.013E-02	Body
cg17616537	*HOXB3*	0.1774	5.839E-03	Body
cg02527112	*HOXD11*	0.1060	1.902E-03	TSS1500
cg20124587	*HOXD11*	0.1081	5.868E-03	TSS1500
cg02470521	*HOXD11*	0.1147	1.379E-02	TSS1500
cg14632140	*LMO3*	0.1055	5.841E-03	5'UTR
cg13572309	*LMO3*	0.1074	5.312E-03	5'UTR
cg01181415	*LMO3*	0.1124	3.887E-03	5'UTR
cg10143811	*LMO3*	0.1351	5.891E-03	5'UTR
cg09526758	*LMO3*	0.1365	7.138E-03	5'UTR
cg01787574	*LMO3*	0.1378	8.395E-03	5'UTR
cg02574073	*LOC404266;HOXB6*	0.1026	4.726E-02	Body;TSS200
cg20184247	*LOC404266;HOXB5*	0.1067	7.639E-03	Body;TSS1500
cg10308785	*LOC404266;HOXB6*	0.2508	7.922E-03	Body;3'UTR
cg26072749	*MIR10A*	0.1075	4.028E-02	Body
cg15649236	*MIR10A*	0.1294	1.075E-02	TSS200
cg14884929	*MIR10A*	0.1342	7.393E-03	TSS1500
cg26916621	*MIR10A*	0.1344	6.323E-03	TSS200
cg04514255	*MIR10A*	0.1578	9.015E-03	TSS1500
cg08297082	*SIM2*	0.1382	1.227E-02	Body
cg27325152	*SIM2*	0.1610	1.164E-02	Body
cg03993743	*SIM2*	0.1658	1.169E-02	Body
cg03760839	*TBX15*	0.1338	2.370E-03	5'UTR
cg24144440	*TBX15*	0.1373	4.442E-03	5'UTR
cg09789768	*TBX15*	0.1633	8.395E-03	5'UTR
cg12664119	*TBX15*	0.1680	1.958E-03	TSS200
cg22378919	*TBX15*	0.1683	1.167E-02	5'UTR
cg06158650	*TBX15*	0.1698	5.906E-03	5'UTR
cg00466334	*TBX15*	0.1722	6.479E-03	TSS1500
cg24842086	*TBX15*	0.1767	2.661E-03	5'UTR
cg02362103	*TBX15*	0.1776	4.837E-03	TSS1500
cg05172122	*TBX15*	0.1826	5.143E-03	5'UTR

cg08942939	*TBX15*	0.1829	6.611E-03	TSS1500
cg23371746	*TBX15*	0.1830	4.442E-03	TSS1500
cg19730691	*TBX15*	0.1874	1.827E-03	5'UTR
cg03942051	*TBX15*	0.1935	3.366E-03	TSS1500
cg26272623	*TBX15*	0.1956	2.067E-03	TSS200
cg22820316	*TBX15*	0.2020	3.391E-03	TSS1500
cg18944010	*TBX15*	0.2120	5.267E-03	5'UTR
cg11391335	*TBX15*	0.2139	5.839E-03	5'UTR
cg13655674	*TBX15*	0.2154	4.785E-03	5'UTR
cg24720355	*TBX15*	0.2461	2.300E-03	5'UTR
cg16990168	*TBX15*	0.2778	2.899E-03	5'UTR
cg25340966	*TBX15*	0.2922	4.346E-03	TSS200
cg14565725	*TBX15*	0.1496	6.323E-03	1stExon;5'UTR
cg07892597	*TBX15*	0.1641	2.067E-03	1stExon;5'UTR
cg24884142	*TBX15*	0.2277	1.827E-03	1stExon;5'UTR
cg10703826	*TBX15*	0.3273	3.370E-03	1stExon;5'UTR
iMT/iLT				
cg13630043	*EMX2*	-0.1758	9.973E-03	3'UTR
cg17320707	*EMX2*	-0.1183	3.455E-02	3'UTR
cg19925204	*EMX2*	-0.1373	6.291E-03	3'UTR;Body
cg20348858	*EMX2;EMX2OS*	-0.1293	1.625E-03	Body;TSS1500
cg10846980	*EMX2;EMX2OS*	-0.1374	1.111E-02	Body;TSS1500
cg15325373	*EMX2;EMX2OS*	0.1105	2.318E-02	1stExon;5'UTR;Body
cg00603371	*EMX2;EMX2OS*	0.1493	3.902E-02	TSS1500;Body
cg06141846	*EMX2OS*	-0.1660	1.722E-02	Body
cg07833262	*EMX2OS*	-0.1307	2.865E-02	Body
cg11124080	*EMX2OS*	-0.1471	1.525E-02	Body
cg12623982	*EMX2OS*	-0.1264	2.809E-02	Body
cg18561589	*EMX2OS*	-0.1675	1.439E-02	Body
cg26342147	*EMX2OS*	-0.1140	9.973E-03	Body
cg20792376	*EMX2OS*	-0.1383	2.621E-02	Body
cg02230593	*EMX2OS*	-0.1298	3.638E-02	Body
cg03311684	*EMX2OS*	-0.1615	8.613E-03	Body
cg04421971	*EMX2OS*	-0.1070	3.031E-02	Body

cg08406102	*EMX2OS*	-0.1397	1.662E-02	Body
cg11195065	*EMX2OS*	-0.1548	1.397E-02	Body
cg16234557	*EMX2OS*	-0.1340	2.151E-02	Body
cg18972885	*EMX2OS*	-0.1320	2.185E-02	Body
cg26288991	*EMX2OS*	-0.1075	3.352E-02	Body
cg13172549	*HOXA3*	0.1006	7.015E-03	5'UTR;TSS200
cg00040268	*HOXA3*	0.1456	3.030E-03	5'UTR
cg00921266	*HOXA3*	0.1128	3.364E-03	5'UTR;TSS200
cg07061298	*HOXA3*	0.1447	1.625E-03	5'UTR;TSS1500
cg07917150	*HOXA3*	0.1539	1.547E-02	5'UTR;TSS1500
cg07942135	*HOXA3*	0.1355	4.381E-03	5'UTR;TSS1500
cg08101036	*HOXA3*	0.1433	1.407E-02	5'UTR;TSS200
cg08164294	*HOXA3*	0.1248	2.154E-02	5'UTR;TSS1500
cg10794257	*HOXA3*	0.1451	1.525E-02	5'UTR;TSS1500
cg26127662	*HOXA3*	0.2267	2.790E-03	5'UTR;TSS1500
cg25768734	*HOXA3*	0.1174	3.820E-03	5'UTR
cg22962123	*HOXA3*	0.1045	9.940E-03	5'UTR;1stExon
cg24900666	*HOXB1*	0.1079	1.830E-02	TSS200
cg26687072	*HOXB1*	0.1045	3.031E-02	TSS200
cg02497558	*HOXB1*	0.1315	5.490E-03	TSS1500
cg08076955	*HOXB1*	0.1001	8.501E-03	TSS200
cg13752649	*HOXB1*	0.1281	9.505E-03	TSS1500
cg24948406	*HOXB1*	0.1027	1.778E-02	5'UTR;1stExon
cg05387167	*HOXB3*	0.1730	4.921E-03	Body
cg19986012	*HOXB3*	0.1182	7.149E-03	Body
cg02458062	*HOXB3*	0.1878	4.025E-03	Body
cg26622699	*HOXB3*	0.1252	7.819E-03	Body
cg10165801	*HOXB3*	0.1424	2.882E-03	5'UTR
cg09952002	*HOXB3*	0.1345	1.588E-02	5'UTR
cg02873421	*HOXB3*	0.1432	7.135E-03	Body
cg15065049	*HOXB3*	0.1249	4.381E-03	Body
cg16787431	*HOXB3*	0.1524	7.135E-03	Body
cg17616537	*HOXB3*	0.1825	3.664E-03	Body
cg21853871	*HOXB3*	0.1305	9.973E-03	Body

cg12570134	*HOXB3*	0.1043	3.423E-02	5'UTR
cg01593673	*HOXB3*	0.1299	4.381E-03	5'UTR
cg02749463	*HOXB3*	0.1164	3.338E-02	5'UTR
cg04800503	*HOXB3*	0.1465	2.406E-03	5'UTR
cg06186155	*HOXB3*	0.1250	6.276E-03	5'UTR
cg23014425	*HOXB3*	0.1219	3.848E-03	5'UTR
cg07850987	*HOXB3*	0.1598	1.341E-02	5'UTR
cg19104015	*HOXB3*	0.1265	2.865E-02	5'UTR
cg25032089	*HOXB3*	0.1146	2.271E-02	5'UTR
cg06395298	*HOXB3*	0.1608	2.271E-03	5'UTR;1stExon
cg12910797	*HOXB3*	0.1041	3.308E-02	5'UTR;1stExon
cg22053945	*HOXB3*	0.1123	2.820E-03	5'UTR;1stExon
cg07080050	*HOXC4;HOXC5;HOXC6*	0.1838	3.958E-04	5'UTR;Body
cg19164987	*HOXC4;HOXC5;HOXC6*	0.1056	3.820E-03	5'UTR;Body
cg08106887	*HOXC4;HOXC5;HOXC6*	0.1172	3.078E-03	5'UTR;Body
cg15772924	*HOXC4;HOXC5;HOXC6*	0.1900	1.948E-04	5'UTR;Body
cg20184247	*LOC404266*	0.1030	1.506E-02	Body;TSS1500
cg09601584	*LOC404266*	0.1148	3.246E-03	Body
cg10308785	*LOC404266*	0.2264	1.890E-02	Body;3'UTR
cg15908709	*LOC404266*	0.1005	2.406E-03	Body;5'UTR
cg04514255	*MIR10A*	0.1731	7.199E-03	TSS1500
cg14884929	*MIR10A*	0.1526	1.625E-03	TSS1500
cg15649236	*MIR10A*	0.1402	3.246E-03	TSS200
cg26916621	*MIR10A*	0.1560	1.895E-03	TSS200
cg10122865	*OTX1*	-0.1139	1.455E-02	3'UTR
cg21472506	*OTX1*	-0.2082	8.002E-03	3'UTR
cg23229261	*OTX1*	-0.2367	5.495E-03	3'UTR
cg04937416	*PTPRN2*	-0.1807	8.740E-03	Body
cg15572489	*PTPRN2*	-0.1562	1.547E-02	Body
cg19241327	*PTPRN2*	-0.1068	3.404E-02	Body
cg10720723	*PTPRN2*	-0.1227	4.191E-02	Body
cg26295921	*PTPRN2*	-0.1231	3.088E-02	Body
cg03993743	*SIM2*	-0.1681	3.345E-02	Body
cg08297082	*SIM2*	-0.1333	1.230E-02	Body

cg27325152	*SIM2*	-0.1325	2.666E-02	Body
cg25340966	*TBX15*	0.2174	3.916E-03	TSS200
cg05172122	*TBX15*	0.1119	4.925E-02	5'UTR
cg24842086	*TBX15*	0.1465	1.350E-03	5'UTR
cg11391335	*TBX15*	0.1287	1.823E-02	5'UTR
cg16990168	*TBX15*	0.1456	1.140E-02	5'UTR
cg24720355	*TBX15*	0.1282	1.813E-02	5'UTR
cg12664119	*TBX15*	0.1125	3.030E-03	TSS200
cg26272623	*TBX15*	0.1241	4.381E-03	TSS200
cg02362103	*TBX15*	0.1019	2.045E-02	TSS1500
cg03942051	*TBX15*	0.1176	1.322E-02	TSS1500
cg08942939	*TBX15*	0.1110	4.431E-02	TSS1500
cg18944010	*TBX15*	0.1223	3.404E-02	5'UTR
cg22820316	*TBX15*	0.1273	7.057E-03	TSS1500
cg07892597	*TBX15*	0.1321	2.406E-03	1stExon;5'UTR
cg10703826	*TBX15*	0.2593	2.982E-03	1stExon;5'UTR
cg24884142	*TBX15*	0.1555	1.600E-03	1stExon;5'UTR

Supplementary table 5: Correlation between the expression and the methylation of each DMP for *TBX15*.

**CpG probe ID**	**Spearman correlation**	**p value**	**Mean ∆β**	**CpG location**
cg08942939	-0.4273	0.0047	0.1829	TSS1500
cg03760839	-0.4322	0.0042	0.1338	5'UTR
cg00466334	-0.4332	0.0042	0.1722	TSS1500
cg24144440	-0.4438	0.0034	0.1373	5'UTR
cg10703826	-0.4445	0.0033	0.3273	1stExon;5'UTR
cg23371746	-0.4541	0.0027	0.1830	TSS1500
cg03942051	-0.4808	0.0015	0.1935	TSS1500
cg05172122	-0.4811	0.0015	0.1826	5'UTR
cg02362103	-0.4826	0.0014	0.1776	TSS1500
cg14565725	-0.4855	0.0013	0.1496	1stExon;5'UTR
cg25340966	-0.504	0.0009	0.2922	TSS200
cg26272623	-0.5045	0.0009	0.1956	TSS200

cg12664119	-0.5135	0.0007	0.1680	TSS200
cg22378919	-0.5135	0.0007	0.1683	5'UTR
cg22820316	-0.5151	0.0007	0.2020	TSS1500
cg24884142	-0.5174	0.0006	0.2277	1stExon;5'UTR
cg16990168	-0.5194	0.0006	0.2778	5'UTR
cg07892597	-0.5248	0.0005	0.1641	1stExon;5'UTR
cg24842086	-0.5266	0.0005	0.1767	5'UTR
cg13655674	-0.5282	0.0005	0.2154	5'UTR
cg19730691	-0.5331	0.0004	0.1874	5'UTR
cg09789768	-0.5441	0.0003	0.1633	5'UTR
cg11391335	-0.5477	0.0003	0.2139	5'UTR
cg18944010	-0.5516	0.0002	0.2120	5'UTR
cg06158650	-0.5555	0.0002	0.1698	5'UTR
cg24720355	-0.5964	<0.0001	0.2461	5'UTR
***Combined**	**-0.5228**	**0.0005**		

* The result of all the DMPs combined.

Supplementary table 6. DMGs showing altered expression in iMT/oLT.

Gene	Expression fold change	Mean Δβ	Relation to Gene	Location
PAX1	-4.3	0.1014 ~ 0.1133	5'UTR, TSS1500	S_Shore, Island
LYPD1	-2.5	-0.1002	1stExon;5'UTR	S_Shore
ALX4	-2.3	0.1001 ~0.1242	TSS1500;3'UTR; Body	S_Shore; Island
HMCN1	-2.2	-0.1299	TSS1500	OpenSea
TBX3	-2.0	-0.2699 ~ -0.1652	5'UTR	Island
EMX2	2.2	-0.1878 ~ -0.1024	Body; TSS1500	N_Shelf; S_Shore
HEPACAM	2.5	0.1422	TSS1500	Island
DLEC1	2.6	-0.1007	Body	N_Shore
EMX2OS	2.8	-0.1723 ~ -0.1039	TSS1500; TSS200; Body; 3'UTR	N_Shore
MAL2	3.9	0.1010	TSS200	Island
LMO3	6.2	0.1074 ~ 0.1378	5'UTR; TSS200, Body	OpenSea

Expression dataset was adopted from the previous study [1].

Supplementary table 7: Shared DMPs in iMT/oLT identified in subchondral bone and the site-matched cartilage.

CpG probe ID	UCSC_RefGene_Name	cartilage_mean Δβ	subchon_mean Δβ
cg19346371	*TBX3*	-0.2485	-0.2699
cg26389913	/	-0.2302	-0.1553
cg08193273	*TRHR*	-0.2075	-0.1110
cg15526081	/	-0.1922	-0.2232
cg20597409	*SLC25A21; LOC100129794*	-0.1914	-0.1291
cg24573321	*BAI3*	-0.1912	-0.1253
cg24725789	/	-0.1889	-0.1561
cg25793931	/	-0.1869	-0.1502
cg12117227	*EXOC2*	-0.1779	-0.1269
cg27630311	*TBX3*	-0.1750	-0.1652
cg16234557	*EMX2OS*	-0.1732	-0.1477
cg03321133	*HOXD8*	-0.1647	-0.1277
cg07774193	/	-0.1640	-0.1134
cg13445608	/	-0.1639	-0.1486
cg08451832	/	-0.1600	-0.1609
cg18784943	/	-0.1600	-0.1529
cg13630043	*EMX2*	-0.1592	-0.1878
cg06896987	/	-0.1577	-0.1506
cg04522596	/	-0.1567	-0.1103
cg06141846	*EMX2OS*	-0.1567	-0.1723
cg03311684	*EMX2OS*	-0.1542	-0.1586
cg02230593	*EMX2OS*	-0.1520	-0.1325
cg25316429	*EMX2OS*	-0.1502	-0.1039
cg04256864	*EMX2OS*	-0.1490	-0.1361
cg11195065	*EMX2OS*	-0.1474	-0.1536
cg11248896	/	-0.1441	-0.1470
cg19925204	*EMX2*	-0.1435	-0.1375
cg09075968	*PCID2*	-0.1434	-0.1176
cg17320707	*EMX2*	-0.1426	-0.1290

cg12777520	*LMX1B*	-0.1370	-0.1091
cg17222143	*USH2A*	-0.1368	-0.1103
cg06456154	/	-0.1362	-0.1296
cg08406102	*EMX2OS*	-0.1357	-0.1623
cg18561589	*EMX2OS*	-0.1352	-0.1658
cg09307788	/	-0.1348	-0.1206
cg25698741	/	-0.1341	-0.1139
cg11958666	/	-0.1336	-0.1039
cg10595547	/	-0.1333	-0.1153
cg12623982	*EMX2OS*	-0.1328	-0.1441
cg26342147	*EMX2OS*	-0.1325	-0.1213
cg18972885	*EMX2OS*	-0.1311	-0.1341
cg07833262	*EMX2OS*	-0.1290	-0.1324
cg20792376	*EMX2OS*	-0.1271	-0.1431
cg19048251	/	-0.1251	-0.1224
cg10846980	*EMX2;EMX2OS*	-0.1246	-0.1486
cg26288991	*EMX2OS*	-0.1239	-0.1083
cg04421971	*EMX2OS*	-0.1238	-0.1278
cg09862711	/	-0.1211	-0.1200
cg11124080	*EMX2OS*	-0.1195	-0.1369
cg00865429	*CLNK*	-0.1159	-0.1058
cg25423004	*HOXD8*	-0.1018	-0.1401
cg21156438	/	0.1018	0.1149
cg24900666	*HOXB1*	0.1031	0.1025
cg08101036	*HOXA3*	0.1044	0.1568
cg14285150	/	0.1066	0.1345
cg22231101	*JAKMIP1*	0.1066	0.1058
cg10165801	*HOXB3*	0.1084	0.1142
cg13777513	/	0.1085	0.1117
cg02337614	/	0.1098	0.1018
cg00875849	/	0.1116	0.1036
cg10399005	/	0.1147	0.1118
cg03942051	*TBX15*	0.1167	0.1935
cg18825414	*C18orf2*	0.1168	0.1159

cg21207665	*PAX9*	0.1169	0.1175
cg20784391	/	0.1186	0.1065
cg20475486	/	0.1197	0.1086
cg04800503	*HOXB3*	0.1201	0.1396
cg02143743	*AGBL1*	0.1204	0.1047
cg05387167	*HOXB3*	0.1206	0.1685
cg22820316	*TBX15*	0.1230	0.2020
cg02362103	*TBX15*	0.1232	0.1776
cg06439547	*COG2*	0.1269	0.1107
cg19975849	/	0.1272	0.1002
cg06982190	/	0.1300	0.1153
cg26127662	*HOXA3*	0.1312	0.2011
cg02873421	*HOXB3*	0.1317	0.1364
cg23143210	/	0.1324	0.1589
cg23348270	/	0.1327	0.1184
cg25332502	*MBP;MBP*	0.1371	0.1207
cg19117047	/	0.1372	0.1110
cg26611765	/	0.1419	0.1155
cg15226608	*PEX5L*	0.1420	0.1103
cg03463818	*TMEM67*	0.1439	0.1104
cg24854430	*ENPP6*	0.1440	0.1493
cg02394746	/	0.1467	0.1197
cg18351329	/	0.1470	0.1519
cg25877512	/	0.1492	0.1235
cg21608519	/	0.1515	0.1348
cg02508651	/	0.1525	0.1444
cg16787431	*HOXB3*	0.1535	0.1533
cg19094333	/	0.1578	0.1241
cg23371746	*TBX15*	0.1587	0.1830
cg10426234	/	0.1589	0.1622
cg02458062	*HOXB3*	0.1598	0.1631
cg02170386	/	0.1615	0.1321
cg16551520	/	0.1617	0.1486
cg19728226	/	0.1644	0.1273

cg26411222	/	0.1649	0.1072
cg07133930	/	0.1672	0.1090
cg04903623	/	0.1698	0.1241
cg09952002	*HOXB3*	0.1702	0.1352
cg10584587	*PACRG*	0.1705	0.1032
cg15539318	/	0.1851	0.1447
cg26073844	*GSDMC*	0.1878	0.1627
cg07280807	/	0.1900	0.1582
cg18470839	/	0.1933	0.1455
cg03807298	/	0.1973	0.1228
cg09284655	/	0.1981	0.1646
cg22005990	/	0.2149	0.1576
cg18220920	/	0.2215	0.1505
cg25255850	/	0.2460	0.1687

Supplementary table 8: Demographic information of patients

**Patient ID**	**Gender**	**Age**	**BMI**	**Height (cm)**	**Weight (kg)**	**OA grade**
P01	M	79	27.07	158.5	68.0	KL3
P02	M	55	37.44	171.4	110.0	KL4
P03	F	66	28.72	155.0	69.0	KL3
P04	F	83	23.43	150.4	53.0	KL4
P05	F	79	21.97	157.5	54.5	KL4
P06	F	82	29.57	145.5	62.6	KL4
P07	F	76	29.07	147.8	63.5	KL4
P08	F	71	24.13	159.0	61.0	KL4
P09	F	78	28.48	145.5	60.3	KL4
P10	F	79	29.13	152.0	67.3	KL4
P11	F	64	26.99	161.4	70.3	KL4
P12	F	82	27.41	152.2	63.5	KL4

Supplementary table 9: Primers used in real-time PCR.

Primer	Sequence

EVX1_F	TTCACCCGAGAGCAGATTG
EVX1_R	CCGGTTCTGGAACCACAC
PTPRN2_F	GCCTTCACCTCTGGGAGATT
PTPRN2_R	GAGGGTATGAATCCGTGCTC
TBX3_F	AAAAATAGACAACAACCCTTTTGC
TBX3_R	ACTGCAGGGTGAGCTGTTTT
SHOX2_F	AACGTAGGTGCTTTAAGGATGC
SHOX2_R	GAAAGGACAAGGGCGTCAC
TBX15_F	TAGCTTCTGGAGACACCTGGA
TBX15_R	GCTGAAGTCTTTGCGAATCAC

Reference

1. Chou, C. H. *et al.* Genome-wide expression profiles of subchondral bone in osteoarthritis. *Arthritis Res Ther* **15**, R190, doi:10.1186/ar4380 (2013).

Supplementary figure legends

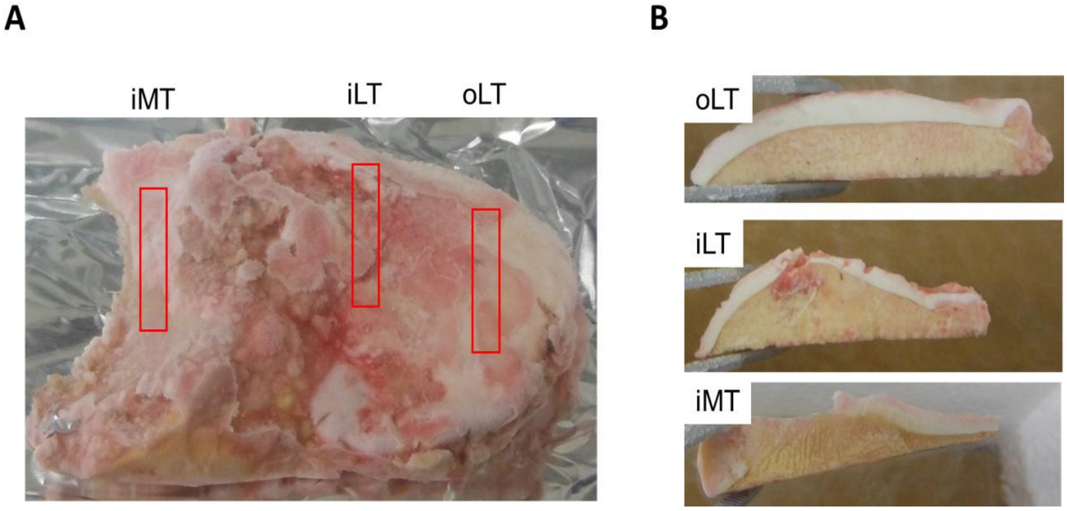

Supplementary figure 1: One representing tibial plateau. (A) The three regions of the tibial plateau, the oLT exhibited macroscopically normal cartilage, the iLT showed intermediate erosion and the iMT had the most severe erosion of the cartilage. (B) The sectional view of the oLT, iLT and iMT regions. oLT: outer region of the lateral tibial plateau, iLT: inner region of lateral tibial plateau, iMT: inner region of medial tibial plateau.

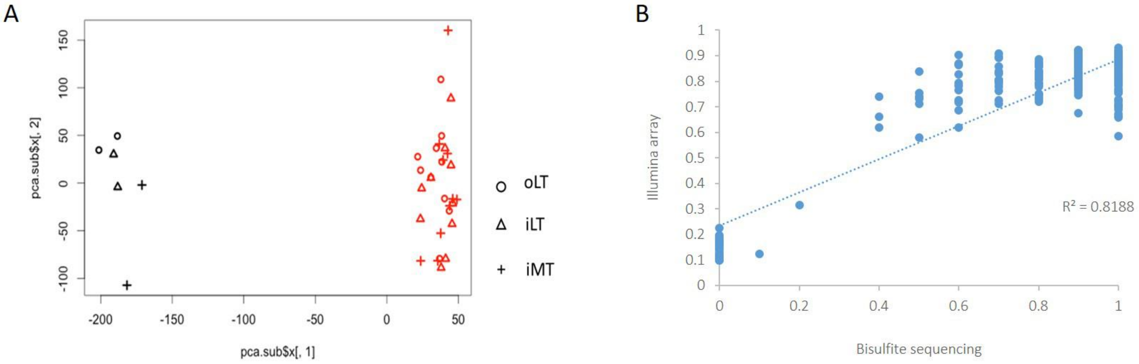

Supplementary figure 2: (A) PCA plot of β values of subchondral bone. "○", "△" and "+" indicate samples from the oLT, iLT and iMT regions, respectively. Red: female, black: male. (B) Correlation between Illumina chip and Bisulfite Sanger sequencing.

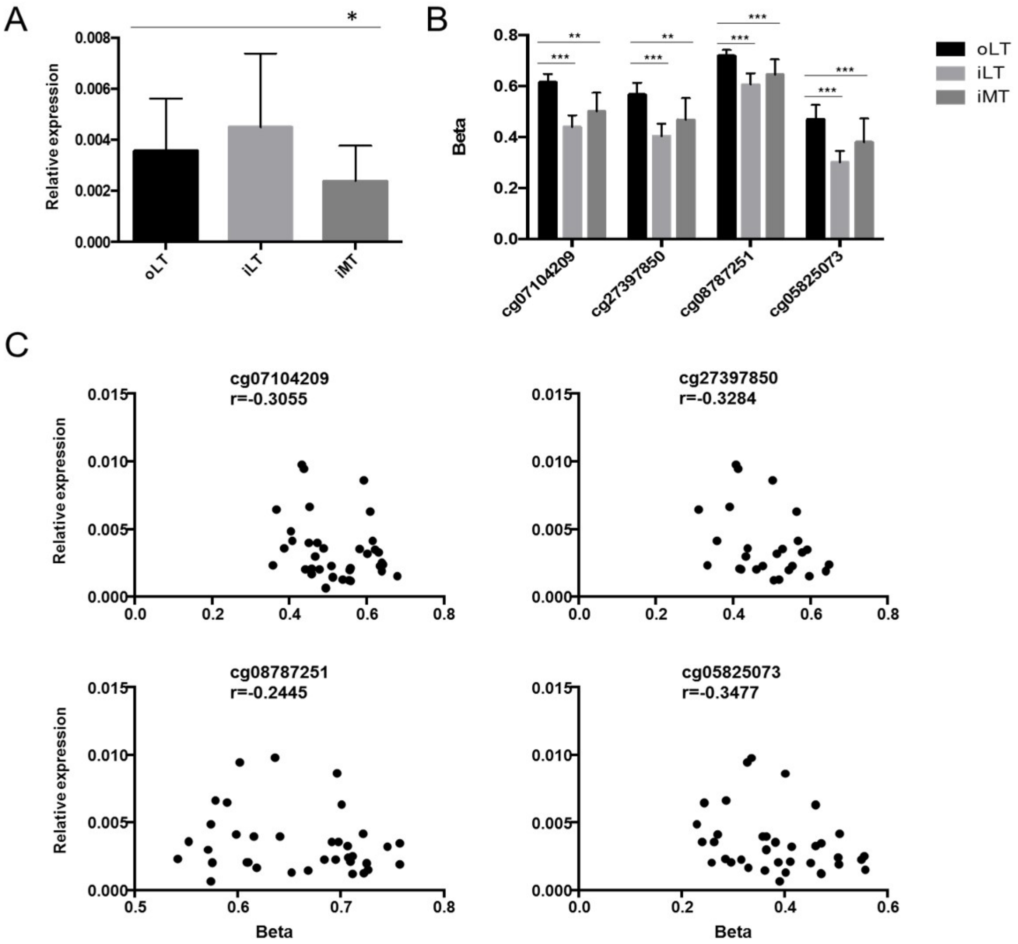

Supplementary figure 3: Relative expression of *EVX1* and the correlation to the methylation of associated DMPs. (A) The relative expression of *EVX1* in subchondral bone of the oLT, iLT and iMT regions. (B) Beta values of the 4 associated CpGs in the subchondral bone of oLT, iLT and iMT regions. (C) Scatter plot showing the correlation between relative gene expression and the methylation beta values. r: the Spearman's rank coefficient. *: p<0.05; **: p<0.01; ***: p<0.001.

A

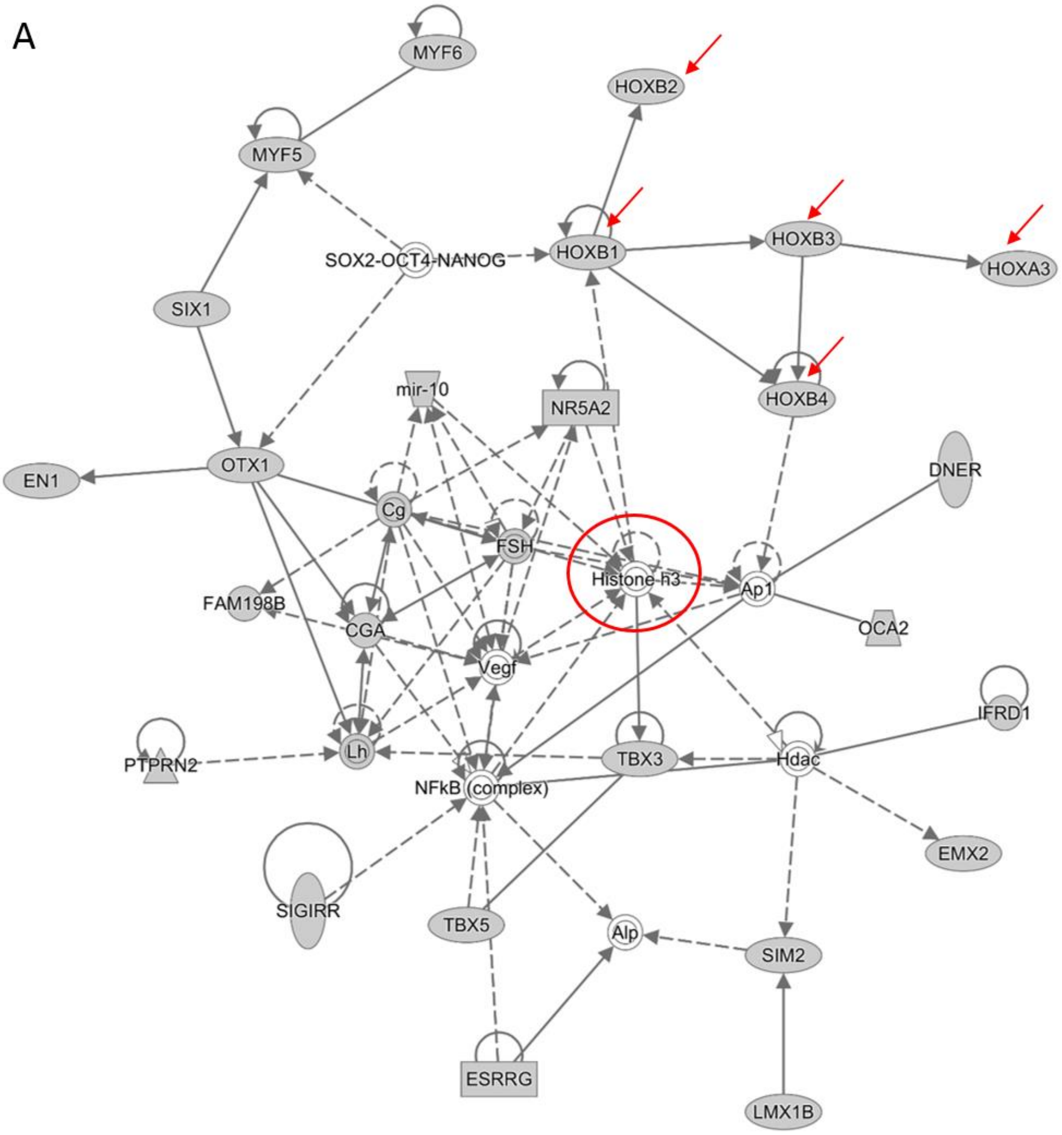
MYF6
HOXB2
MYF5
HOXB1
HOXB3
HOXA3
SOX2-OCT4-NANOG
SIX1
mir-10
NR5A2
HOXB4
EN1
OTX1
DNER
Cg
FSH
Histone h3
Ap1
FAM198B
CGA
OCA2
Vegf
IFRD1
PTPRN2
Lh
TBX3
Hdac
NFkB (complex)
EMX2
SIGIRR
TBX5
Alp
SIM2
ESRRG
LMX1B

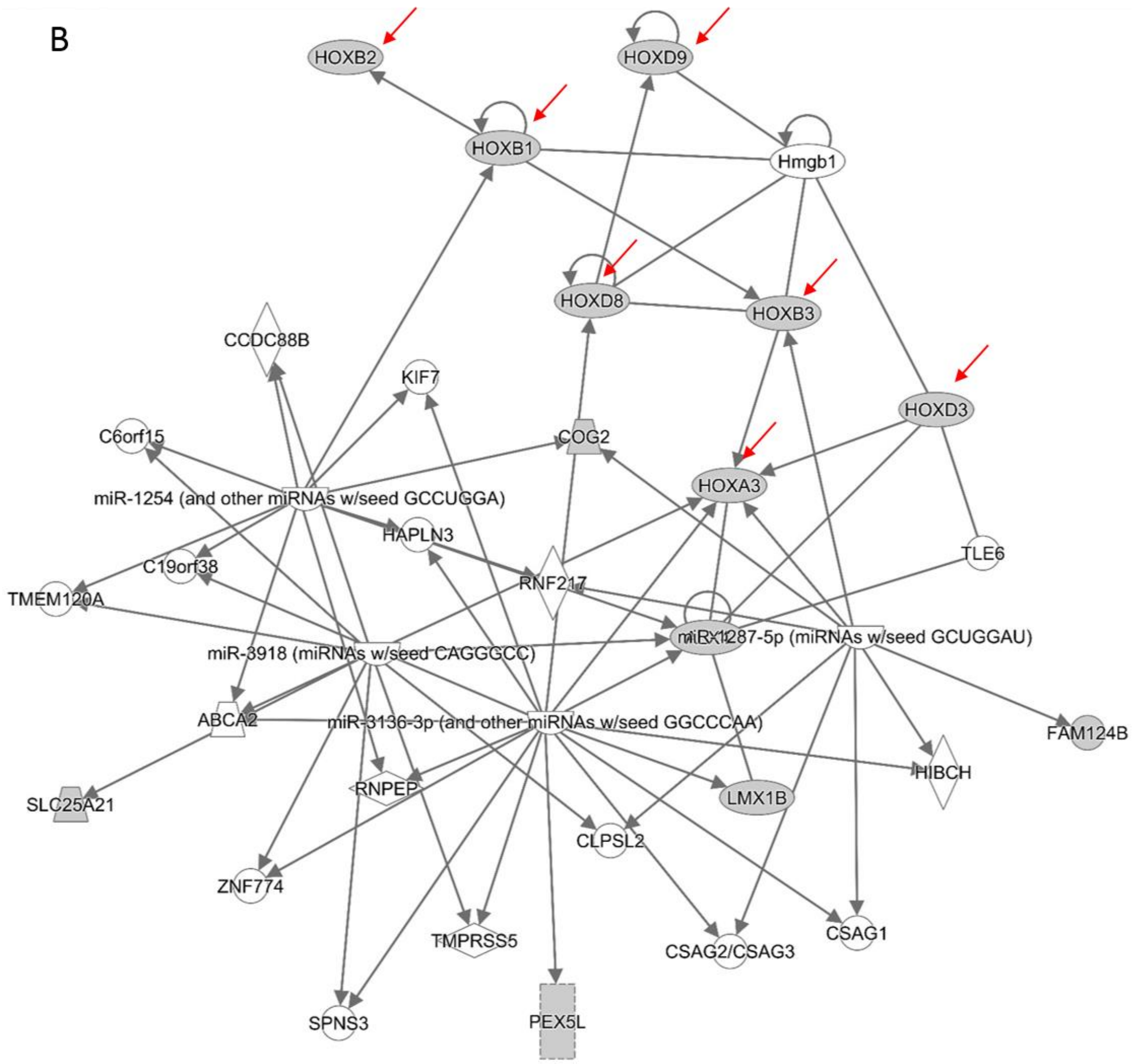
B
HOXB2
HOXD9
HOXB1
Hmgb1
HOXD8
HOXB3
CCDC88B
KIF7
HOXD3
C6orf15
COG2
HOXA3
miR-1254 (and other miRNAs w/seed GCCUGGA)
HAPLN3
TLE6
C19orf38
RNF217
TMEM120A
miR-1287-5p (miRNAs w/seed GCUGGAU)
miR-3918 (miRNAs w/seed CAGGGCC)
ABCA2
miR-3136-3p (and other miRNAs w/seed GGCCCAA)
FAM124B
HIBCH
RNPEP
LMX1B
SLC25A21
CLPSL2
ZNF774
TMPRSS5
CSAG1
CSAG2/CSAG3
SPNS3
PEX5L

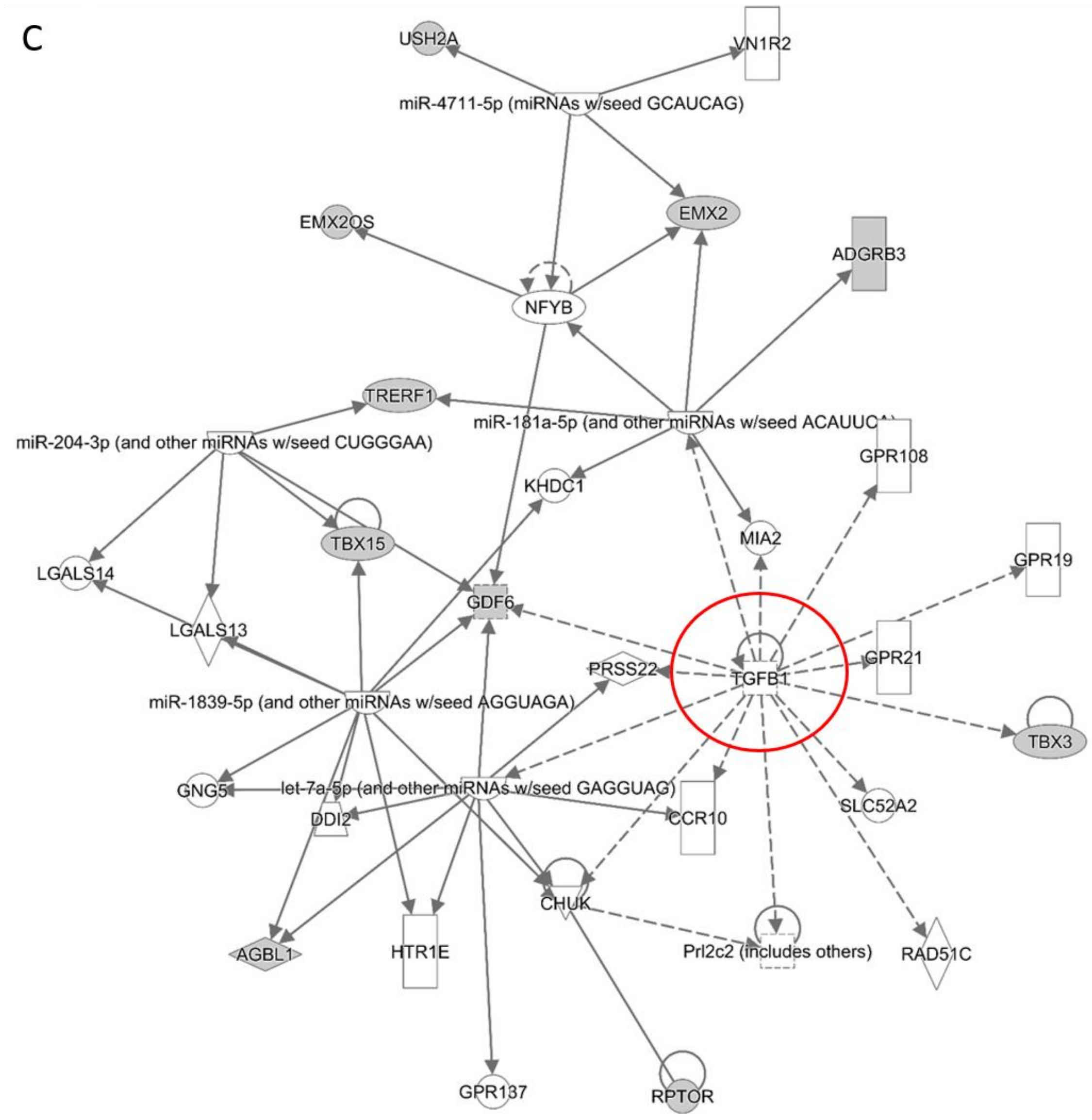

Supplementary figure 4: Predicted networks identified by IPA. (A) Network associated with DMGs in iMT/iLT group of subchondral bone highlighted the HOX transcription factors (red arrows) and centered on histone H3 (red circle). (B) Network 1 associated with shared DMGs identified in subchondral bone and cartilage in iMT/oLT group, which highlighted the cluster of HOX transcription factors (red arrows). (C) Network 2 associated with shared DMGs identified in subchondral bone and cartilage in iMT/oLT group, showed the centered node TGFB (red circle), which was key pathway for both chondrogenesis and bone remodeling.

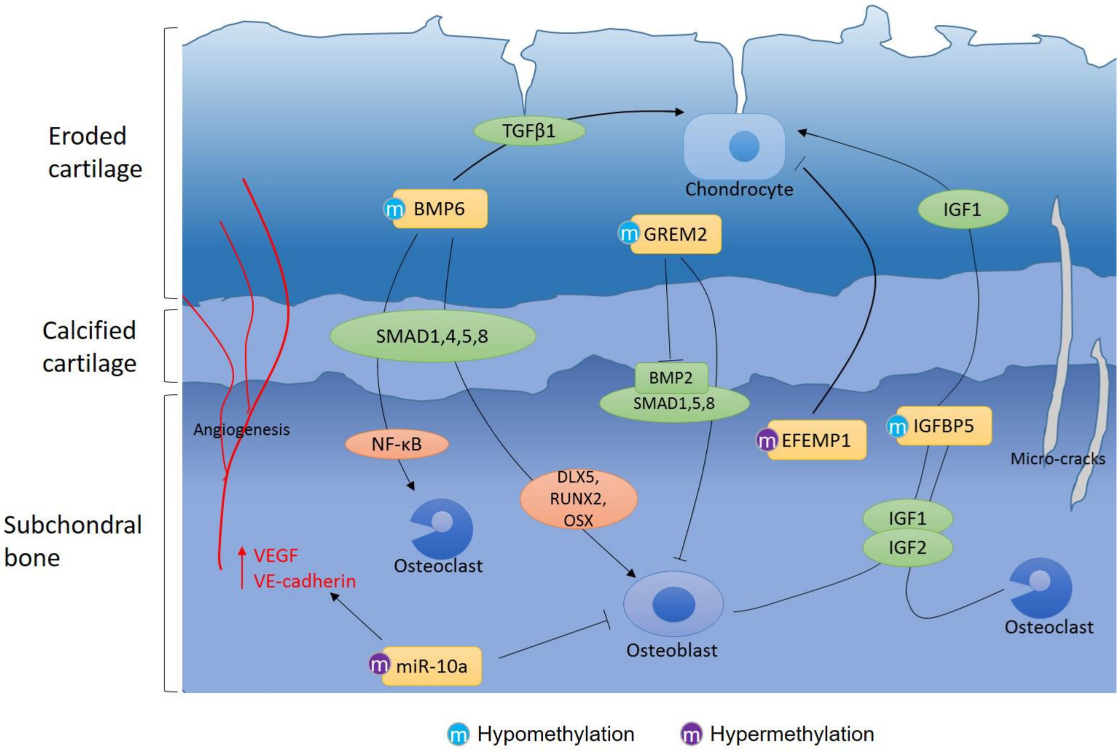

Supplementary figure 5. Example DMGs participated in the molecular crosstalk between the subchondral bone and cartilage at the late stage of OA.
